# Vitamin B12 and Folate in Adherent and Non-Adherent Individuals with Phenylketonuria: A Cross-Sectional Study, Systematic Review, and Meta-Analysis

**DOI:** 10.3390/metabo15070438

**Published:** 2025-07-01

**Authors:** Kamila Bokayeva, Małgorzata Jamka, Dariusz Walkowiak, Monika Duś-Żuchowska, Łukasz Kałużny, Natalia Wichłacz-Trojanowska, Agnieszka Chrobot, Renata Mozrzymas, Gulnara Sultanova, Karl-Heinz Herzig, Jarosław Walkowiak

**Affiliations:** 1 Department of Pediatric Gastroenterology and Metabolic Diseases, Poznan University of Medical Sciences, Szpitalna Str. 27/33, 60-572 Poznań, Poland; kamila.bokayeva@student.ump.edu.pl (K.B.); mjamka@ump.edu.pl (M.J.); mduszuchowska@ump.edu.pl (M.D.-Ż.); lkaluzny@ump.edu.pl (Ł.K.); natalia.wichlacztrojanowska@ump.edu.pl (N.W.-T.); karl-heinz.herzig@oulu.fi (K.-H.H.); 2 Doctoral School, Poznan University of Medical Sciences, Bukowska 70, 60-812 Poznań, Poland; 3 Department of Organization and Management in Health Care, Poznan University of Medical Sciences, Marii Magdaleny Str. 14, 61-786 Poznań, Poland; 4 Metabolic Outpatient Clinic, Voievodship Children Hospital, Chodkiewicza Str. 44, 85-667 Bydgoszcz, Poland; aga.chrobot@wp.pl; 5 Research and Development Center, Regional Specialist Hospital, Department of Pediatrics, Kamienskiego Str. 73a, 51-124 Wrocław, Poland; mozrzymas@wssk.wroc.pl; 6 School of Dentistry, Pharmacy, Nursing, Public Health and Preventive Medicine, West Kazakhstan Marat Ospanov Medical University, Maresyev Str. 68, Aktobe 030019, Kazakhstan; stomfak.zkgmu@mail.ru; 7 Research Unit of Biomedicine and Internal Medicine, Faculty of Medicine, University of Oulu, Aapistie Str. 5, 90220 Oulu, Finland; 8 Biocenter Oulu, University of Oulu, Aapistie Str. 5, 90220 Oulu, Finland; 9 Medical Research Center, Oulu University Hospital, Aapistie Str. 5, 90220 Oulu, Finland

**Keywords:** PKU, inborn errors of metabolism, adherence, vitamins, nutrition, metabolism, diet therapy

## Abstract

**Background/Objectives:** The impact of dietary adherence and regular formula intake on the vitamin levels in individuals with phenylketonuria (PKU) remains unclear. This study aimed to assess the influence of both adherence to dietary management and regular formula intake on the vitamin B12 and folate levels in individuals with PKU. **Methods:** This cross-sectional multicentre study included 63 patients with PKU aged 12–41 years. The participants were classified as adherent or non-adherent based on their mean plasma phenylalanine levels or as regular or irregular formula consumers. The participants’ vitamin B12 and folate levels were compared across these groups. In addition, a systematic search of PubMed, Web of Science, Scopus, and Cochrane Library identified 11,631 studies comparing vitamin B12 and folate levels between adherent vs. non-adherent patients and regular vs. irregular formula intake groups, of which eight met the inclusion criteria. Analyses were conducted using random-effects and fixed-effects models and effect sizes were expressed as standardised mean differences (SMDs). **Results:** This cross-sectional study showed significantly higher vitamin B12 and folate levels in adherent vs. non-adherent individuals (767.6 ± 264.5 vs. 524.7 ± 216.4 pg/mL; 13.44 ± 1.96 vs. 10.62 ± 3.36 ng/mL, both *p* < 0.001) and in regular vs. irregular formula consumers (746.7 ± 228.4 vs. 527.4 ± 281.9 pg/mL; 13.32 ± 2.25 vs. 10.48 ± 3.23 ng/mL, *p* < 0.0001 and *p* < 0.001 respectively). The meta-analysis found no significant differences between the adherent and non-adherent groups, which were defined based on their phenylalanine levels, but showed higher vitamin B12 levels (fixed-effects model, SMD: 1.080, 95% CI: 0.754, 1.405, *p* < 0.0001) and a near-significant trend toward higher folate levels (random-effects model, SMD: 0.729, 95% CI: −0.032, 1.490, *p* = 0.061) in regular formula consumers. **Conclusions:** Regular formula intake is a key determinant of vitamin B12 in patients with PKU. These findings highlight the importance of consistent formula use in dietary management and warrant further research.

## 1. Introduction

Phenylketonuria (PKU) is a rare inherited metabolic disorder that is characterised by a deficiency in the enzyme phenylalanine hydroxylase (PAH), which is crucial for the conversion of phenylalanine (Phe) to tyrosine. This enzymatic deficiency leads to phenylalanine accumulation in the blood, potentially resulting in severe neurological and psychiatric impairments if not managed appropriately [1].

PKU has a global prevalence of approximately 1 in 23,930 live births, with a notably higher prevalence in Poland, where it occurs in ~1 in 8309 live births [2]. The cornerstone of PKU management involves a strict lifelong dietary restriction of Phe, supplemented with specialised medical formulas designed to provide necessary nutrients, i.e., vitamins, minerals, and trace elements, while limiting Phe intake [3]. Adherence to the dietary regimen is critical to preventing the neurotoxic effects of elevated Phe levels, but this is challenging for many patients, particularly as they age [4,5].

Despite the increased availability of medical foods, specialised low-protein products, and approved medications, many adolescents and older patients with PKU continue to struggle to maintain the recommended Phe levels [4,6,7,8] due to an insufficient understanding of dietary restrictions, low motivation, difficulties in coping with the disease [9], the taste and texture of Phe-free foods, and the cost of specialised low-protein products [10], as well as psychosocial and emotional issues, family dynamics, parental involvement, attitudes toward healthcare providers, and, in some cases, a lack of reimbursement for special dietary foods [11,12]. Managing a highly restrictive diet is a significant aspect of life for individuals with PKU, and often leads to disordered eating patterns. Disordered eating refers to a range of irregular eating behaviours, including guilt and shame associated with eating, preoccupation with food, frequent dieting, anxiety around certain foods, and loss of control over eating. Some individuals may also avoid eating in public due to past experiences of being bullied about their diet [3]. While non-adherence is clearly linked to elevated Phe levels and potential cognitive decline, its impact on other nutritional parameters, particularly vitamin status, remains less well understood.

Our previous meta-analysis [13] showed that individuals with PKU have significantly higher folate concentrations than healthy controls, with patients with PKU aged 18 years and older having higher vitamin B12 levels than healthy individuals. However, these findings may have been influenced by various factors, with variability in adherence to treatment regimens among patients with PKU being one of them. Folate deficiency in PKU seems to be rare [14], although it has been occasionally found in some patients [15]. However, elevated plasma folate levels are frequently reported, particularly in children and adolescents adhering to a dietary regimen [16,17]. Vitamin B12 data show greater variability. Ahmadzadeh et al. [15], in a group of 75 continuously treated patients with PKU, found eight subjects who exhibited low vitamin B12 levels, while 28 participants showed elevated concentrations. Another study reported vitamin B12 deficiency in 15% of 112 patients [18]. One study showed no vitamin B12 deficiency among patients who were maintaining well-controlled mean Phe levels, which suggests that adherence plays a key role in preventing deficiencies [19]. Schulpis et al. [20] compared adherent and non-adherent patients with PKU and observed reduced levels of vitamin B12 and folate in those who had poorly controlled mean Phe levels. Two studies demonstrated that the vitamin B12 levels were significantly lower, respectively, in subjects not consuming amino acid mixture and non-adherent patients [21,22], although one of them noted that the levels remained within the normal range in both groups [21].

Vitamin B12 and folate are essential nutrients with significant roles in neurological and hematological health. Given the variability in reported vitamin levels, it is worth systematically examining how dietary adherence affects these levels, as this has not been thoroughly studied yet. Therefore, the present cross-sectional study, which is followed by a systematic review and meta-analysis, aims to compare the vitamin B12 and folate levels in adherent versus non-adherent individuals with PKU. Since the definition of adherence was not uniform across the analysed studies, we distinguished two major classifications that were used by authors. We separately analysed patients with higher vs. lower Phe levels and individuals who regularly took the formula vs. those who did not. It was hypothesised that the vitamin B12 and folate status in adherent individuals do not differ from those of non-adherent patients, as well as that regular formula consumers will not differ from irregular consumers.

## 2. Cross-Sectional Study

This cross-sectional observational study was reported in accordance with the Strengthening the Reporting of Observational Studies in Epidemiology (STROBE) guidelines [23,24]. The guidelines provide a checklist of items that should be included in reports of observational studies, including cross-sectional studies, to ensure transparency and completeness.

### 2.1. Materials and Methods

#### 2.1.1. Study Design and Participants

In total, 63 patients with PKU of both sexes who were aged between 12 and 41 years were recruited from three reference centres for PKU, the Department of Pediatric Gastroenterology and Metabolic Diseases of the Poznan University of Medical Sciences (Poznan, Poland), the Research and Development Centre, Regional Specialist Hospital (Wrocław, Poland), and Voievodship Children Hospital (Bydgoszcz, Poland). The minimum sample size was calculated using the mean and standard deviation values reported in a previous study that assessed the vitamin B12 and folate levels in adherent and non-adherent patients with PKU [25]. The difference in means was assumed to be 100 pmol/L for vitamin B12. For two independent samples [26], the required sample size to detect this difference with 80% power and 95% confidence was calculated as 25 participants per group. To account for potential dropouts (estimated at 10%), it was determined that at least 56 patients should be recruited. Recruitment occurred from June 2024 to December 2024, with data on relevant baseline characteristics being collected at enrolment. The inclusion criteria were as follows: participants aged ≥12 years, diagnosed with classic PKU with a pre-treatment Phe concentration >1200 μmol/L, identified through a newborn screening programme, and on continuous treatment. Additionally, the participants needed to be over 12 years old, and they and their parents needed to be willing to participate in the study. The exclusion criteria were as follows: participants with a chronic or acute disease that could impact PKU treatment or vitamin absorption and metabolism, those undergoing treatment with tetrahydrobiopterin (BH4) or pegvaliase, those who were supplemented with folate or vitamin B12, and those who were pregnant or breastfeeding.

The data collected included the body mass index (BMI), mean and median plasma Phe concentrations, percentages of abnormal values, serum folate, and vitamin B12 levels. The BMI was calculated by dividing the patient’s weight in kilograms by the square of their height in meters. For participants ≤18 years of age, the BMI was recalculated using the International Obesity Task Force values (BMI-IOTF corrected) [27]. Mean and median plasma Phe concentrations were collected from clinical records for the past 24 months.

Participants were categorized into two groups based on their mean annual plasma Phe levels. High plasma Phe levels (non-adherent group) were defined as a mean plasma Phe concentration >600 μmol/L (>10 mg/dL), while low plasma Phe levels (adherent group) were defined as ≤600 μmol/L (≤10 mg/dL). This threshold aligns with clinical guidelines for optimal metabolic control in patients with PKU who are older than 12 years of age [28].

Additionally, the participants were divided based on their formula intake, into regular and irregular formula intake groups. Regular formula intake was defined as consuming the prescribed amount of formula consistently across meals and days, as documented in clinical records and dietary interviews, while irregular intake was defined as inconsistent or incomplete adherence to the prescribed formula consumption (less than two-thirds of the recommended intake).

#### 2.1.2. Vitamin Assessment

Vitamin B12 and folate levels were quantified by a chemiluminescent method at a commercial laboratory (ALAB), with the assessments being performed on the Alinity i system analyser (Abbott Laboratories, Abbott Park, IL, USA). Vitamin B12 levels were assessed with the reference range provided by the laboratory, which was set at 187–883 pg/mL. Folate levels were measured with the laboratory reference ranges outlined in Table 1. All biochemical measurements were obtained from fasting morning blood samples. All collected data were anonymized and stored securely in compliance with ethical guidelines and institutional policies. The laboratory personnel conducting the analyses were blinded to all study-related information. The group allocation was performed only after all laboratory analyses were completed, to ensure an independent and unbiased assessment.

#### 2.1.3. Ethics Approval and Consent to Participate

The study protocol was approved by the Poznan University of Medical Sciences Ethical Committee (approval number: 260/24, date of approval: 10 April 2024), and it was conducted according to the principles of the Helsinki Declaration [29]. All patients or their parents (for children under 18 years) were properly informed and asked to sign an informed consent form to participate in the study.

#### 2.1.4. Statistical Analysis

The study variables were characterised using the medians with interquartile ranges (IQR) and the means with standard deviations (SD). The 95% confidence interval (95% CI) was also reported for the vitamin results. The normality of the variables was assessed using the Shapiro–Wilk test, with the Mann–Whitney test being performed for non-normally distributed data. Levene’s test for homogeneity of variances was used to determine whether the variances of the two groups were equal for the normally distributed data. If the variances were equal, the Student’s *t*-test for independent samples was applied; otherwise, Welch’s *t*-test was used. A Chi-square test was performed to assess whether the proportions of males and females differed between the groups. A *p*-value < 0.05 was considered significant. Statistical analyses were conducted using PQStat (PQStat Software, v.1.8.6. Poznan, Poland) and RStudio (v.2024.12.0+467, Posit Software, PBC, Boston, MA, USA) [30].

#### 2.1.5. Bias Control

Several strategies were applied to mitigate potential sources of bias. Minimising selection bias in a PKU cohort is inherently challenging due to the rarity and unique characteristics of the condition; therefore, as broad a range of patients with PKU as possible was included from the available treatment centres to capture a sample that reflects the diversity within the PKU population. Measurement bias was addressed by utilising validated tools and standardised protocols for data collection. Information bias was reduced through the use of clearly defined variables and reliance on objective measures whenever possible.

### 2.2. Results

**Figure 1:**
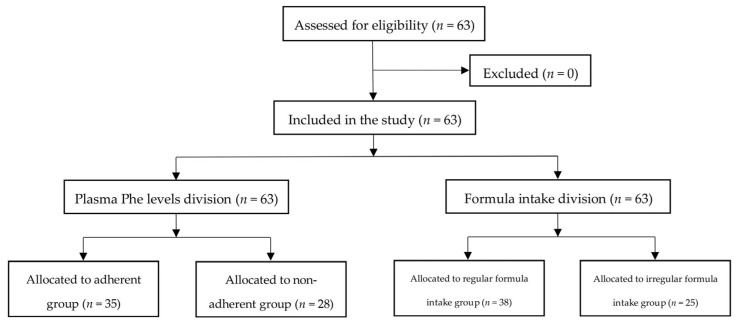
Participants flow diagram.

A total of 63 individuals were initially identified as potentially eligible for this study (Figure 1). After screening, 63 individuals were confirmed to be eligible and invited to participate, with all agreeing to participate and thus being included in the final analysis. No patients were withdrawn or excluded.

The participants were divided into adherent (*n* = 35) and non-adherent (*n* = 28) groups based on their mean plasma Phe levels (Table 2). There were no significant differences in BMI (21.71 ± 3.35 kg/m^2^ vs. 24.78 ± 5.42 kg/m^2^, *p* = 0.067), age (20.88 ± 7.33 vs. 24.01 ± 8.38, *p* = 0.109), or sex (*p* = 0.208) between the groups. Overall, 42.9% (15 participants) of the adherent individuals and 17.9% (5 participants) of the non-adherent individuals exhibited folate levels above the normal range. Twelve (34.3%) adherent individuals had elevated vitamin B12 levels compared to two patients (7.1%) in the non-adherent group. One participant in the non-adherent group had vitamin B12 deficiency.

The mean Phe level was 7.09 ± 2.13 mg/dL in the adherent group, which had 20.9 ± 15.1% abnormal Phe values, compared to 15.62 ± 3.53 mg/dL in the non-adherent group, which had 88.9 ± 14.9% abnormal Phe values. The vitamin B12 and folate levels were significantly higher in the adherent group (767.6 ± 264.5 pg/mL vs. 524.7 ± 216.4 pg/mL, *p* < 0.001; 13.44 ± 1.96 ng/mL vs. 10.63 ± 3.36 ng/mL, *p* < 0.001).

Table 3 compares individuals with regular and irregular formula intake. No significant differences were observed in terms of the age (20.82 ± 7.25 vs. 24.48 ± 8.49 years, *p* = 0.087) or sex distribution (65.8% vs. 52.0% female, *p* = 0.161) between groups. However, the BMI was higher in the irregular group (25.01 ± 5.35 vs. 21.80 ± 3.60 kg/m^2^, *p* = 0.012). Metabolic differences were pronounced, with the regular group showing significantly lower mean Phe levels (8.16 ± 3.17 vs. 15.02 ± 4.76 mg/dL, *p* < 0.0001) and a lower percentage of abnormal Phe values (32.7 ± 30.2% vs. 79.0 ± 28.7%, *p* < 0.0001). The mean vitamin B12 (746.7 ± 228.4 vs. 527.4 ± 281.9 pg/mL, *p* = 0.0001) and folate levels (13.32 ± 2.25 vs. 10.48 ± 3.23 ng/mL, *p* < 0.001) were also significantly higher in the regular intake group. Folate levels above the normal range were more common in the regular group (44.7%, 17 individuals) than in the irregular group (12%, 3 individuals). Similarly, elevated vitamin B12 levels were found in 29% (11 individuals) of the regular group versus 12% (3 individuals) of the irregular group, with one case of deficiency in the latter.

## 3. Systematic Review and Meta-Analysis

### 3.1. Materials and Methods

#### 3.1.1. Protocol and Registration

This meta-analysis followed the Preferred Reporting Items for Systematic Reviews and Meta-Analyses (PRISMA) [31] and the Cochrane [32] guidelines. PRISMA 2020 is an updated guideline that is designed to improve the reporting of systematic reviews, outlining key information that should be clearly presented. The Cochrane Handbook provides comprehensive methodological guidance for conducting systematic reviews and meta-analyses of interventions, particularly of randomised controlled trials. This study was registered in the International Prospective Register of Systematic Reviews (PROSPERO) (CRD420250650808) [33] and the protocol of this meta-analysis can be accessed at the website.

#### 3.1.2. Inclusion and Exclusion Criteria

The search strategy focused exclusively on human studies published in English in peer-reviewed journals. To be eligible for inclusion in the meta-analysis, studies were required to compare the vitamin B12 and/or folate levels between adherent and non-adherent patients with PKU. The eligibility criteria included individuals who were diagnosed with PKU during the neonatal period and managed with early intervention, including a Phe-restricted diet.

##### Division by Mean Phe Levels

Adherence to dietary management in individuals with PKU was defined based on the reported mean plasma Phe levels. The criteria for adherence were determined according to Vockley et al. [28] as follows:

• For patients under 6 years of age, adherence was defined as maintaining a mean plasma Phe level of <360 μmol/L;
• For patients aged 6 to 10 years, a mean plasma Phe level of <480 μmol/L;
• For patients aged 10 years and older, a mean plasma Phe level of ≤600 μmol/L.

Patients whose mean plasma Phe levels exceeded the specified thresholds for their age group were classified as non-adherent. Only studies that directly included or allowed the calculation of adherence and non-adherence based on mean plasma Phe levels were incorporated into the meta-analysis.

##### Division by Formula Consumption

To expand the scope of the analysis, another stratification was performed in this meta-analysis based on consumption of the formula:

• Regular formula intake: participants who consistently consumed the recommended amounts of metabolic formula as part of their dietary regimen;
• Irregular formula intake: participants who reported inconsistent or insufficient consumption of the metabolic formula, as defined in the study protocols or dietary records.

Thereafter, separate analyses were conducted for both division types, with studies older than 20 years being excluded to account for changes in the nutritional composition of formulas over time. This step ensured that the findings reflected the impact of modern formulations. Studies were required to provide detailed, extractable data, including the number of participants, their demographics, and the vitamin B12 and folate measurements for both groups. The exclusion criteria included pregnant or lactating women, mild hyperphenylalaninemia, studies in which participants were additionally supplemented with any of the vitamins under review, and those involving patients with PKU who were treated with tetrahydrobiopterin (BH4) or pegvaliase. Additionally, conference abstracts, abstract-only papers, and publications without full data were excluded from consideration.

#### 3.1.3. Data Collection Process, Extraction and Analysis

Two independent reviewers (K.B. and M.J.) searched each database, following the predefined inclusion and exclusion criteria. The reviewers first assessed the titles, then the abstracts, which was followed by a detailed review of the full texts that was based on the eligibility criteria. Duplicate entries and ineligible studies were excluded. Any study considered relevant by at least one reviewer was advanced to the next stage. If there were any uncertainties or disagreements during the selection process, discrepancies were discussed with a senior researcher (J.W.) to reach a consensus [31]. Each publication was thoroughly evaluated and the study authors were contacted for further clarification if any information was missing, such as statistical data. The articles were compiled and managed using Zotero, an open-source reference management tool (Zotero, version 7.0.11, https://www.zotero.org/, accessed on 17 December 2024). This systematic review presented results for vitamins that were assessed in at least two studies. A quantitative analysis was conducted in the form of a meta-analysis.

#### 3.1.4. Data Item

The extracted information included the following:

1. General information: title of the article, journal name, main author, and publication year;
2. Study characteristics: study name, design, country (region), and sample size (total number of subjects and the number in each group who were included and completed the study);
3. Study population characteristics: age, sex, and BMI (kg/m^2^);
4. Description of dietary treatment: natural protein intake (g/day), protein substitute intake (g/day), total protein intake (g/day), phenylalanine intake (mg/d), annual mean/median phenylalanine levels (μmol/L), follow-up (yes or no), treatment adherence (yes or no), phenylalanine levels (μmol/L), and tyrosine levels (μmol/L);
5. Main outcomes: blood or plasma levels of folate (nmol/L), folic acid (nmol/L), erythrocyte folate (nmol/L), total folate (nmol/L), and vitamin B12 (pmol/L).

#### 3.1.5. Information Sources and Search Strategy

A systematic search was performed in PubMed (Medline), Scopus, Web of Science, and the Cochrane Library for publications from December 2024 to January 2025 to identify studies that provided data on the vitamin B12 and folate blood levels in adherent versus non-adherent patients with PKU. The analysis included both experimental studies (randomised and non-randomised controlled trials) and observational studies (case-control and cross-sectional studies) with no restrictions on publication date. Reference lists from relevant review articles and individual studies were manually searched to identify any potentially missed studies and to ensure comprehensive coverage of the literature. The search term combinations that were used to ensure comprehensive coverage included “PKU”, “phenylketonuria”, “vitamin”, “diet”, and “nutrition”. Key terms were also combined with their synonyms and alternative terminology related to PKU and vitamins. This process included reviewing Medical Subject Headings (MeSH) terms and exploring synonym databases. Moreover, the published articles, systematic reviews, and meta-analyses were screened to ensure a comprehensive search, with additional terms and concepts being incorporated into our search strategy.

The search strategy, in more detail, was as follows:

Cochrane: “phenylketonuria” OR “phenylalanine hydroxylase deficiency” OR “phenylalanine hydroxylase deficient” OR “PKU” OR “hyperphenylalaninaemia” OR “BH4 deficiency” OR “BH4 deficient” OR “tetrahydrobiopterin deficiency” OR “tetrahydrobiopterin deficient” OR “PAH deficiency” OR “PAH deficient” OR “phenylketonuric” OR “hyperphenylalaninaemic” in Title Abstract Keyword AND “dietary” OR “supplement” OR “supplementations” OR “supplementation” OR “nutritional” OR “nutrition” OR “diet” OR “diets” OR “vitamin” OR “vitamins” OR “vitaminization” OR “vitaminisation” OR “nutrient” OR “nutrients” OR “micronutrient” OR “micronutrients” OR “water soluble” OR “water-soluble” OR “folic” OR “folate” OR “folacin” OR “pteroylglutamic” OR “Pteroyl-L-glutamate” OR “Pteroyl-L-glutamic” OR “cobalamin” OR “cobalamins” OR “cobalamine” OR “cobalamines” OR “cyanocobalamin” OR “cyanocobalamine” in the title, abstract and keywords sections—(January 2025).

PubMed: (“phenylketonuria” OR “phenylalanine hydroxylase deficiency” OR “phenylalanine hydroxylase deficient” OR “PKU” OR “hyperphenylalaninaemia” OR “BH4 deficiency” OR “BH4 deficient” OR “tetrahydrobiopterin deficiency” OR “tetrahydrobiopterin deficient” OR “PAH deficiency” OR “PAH deficient” OR “phenylketonuric” OR “hyperphenylalaninaemic” [MeSH Terms]) AND (“dietary” OR “supplement” OR “supplementations” OR “supplementation” OR “nutritional” OR “nutrition” OR “diet” OR “diets” OR “vitamin” OR “vitamins” OR “vitaminization” OR “vitaminisation” OR “nutrient” OR “nutrients” OR “micronutrient” OR “micronutrients” OR “water soluble” OR “water-soluble” OR “folic” OR “folate” OR “folacin” OR “pteroylglutamic” OR “pteroyl-L-glutamate” OR “Pteroyl-L-glutamic” OR “cobalamin” OR “cobalamins” OR “cobalamine” OR “cobalamines” OR “cyanocobalamin” OR “cyanocobalamine” [MeSH Terms])—(January 2025).

Scopus: (TITLE-ABS-KEY (“phenylketonuria” OR “phenylalanine hydroxylase deficiency” OR “phenylalanine hydroxylase deficient” OR “PKU” OR “hyperphenylalaninaemia” OR “BH4 deficiency” OR “BH4 deficient” OR “tetrahydrobiopterin deficiency” OR “tetrahydrobiopterin deficient” OR “PAH deficiency” OR “PAH deficient” OR “phenylketonuric” OR “hyperphenylalaninaemic”) AND TITLE-ABS-KEY (“dietary” OR “supplement” OR “supplementations” OR “supplementation” OR “nutritional” OR “nutrition” OR “diet” OR “diets” OR “vitamin” OR “vitamins” OR “vitaminization” OR “vitaminisation” OR “nutrient” OR “nutrients” OR “micronutrient” OR “micronutrients” OR “water soluble” OR “water-soluble” OR “folic” OR “folate” OR “folacin” OR “pteroylglutamic” OR “pteroyl-l-glutamate” OR “pteroyl-l-glutamic” OR “cobalamin” OR “cobalamins” OR “cobalamine” OR “cobalamines” OR “cyanocobalamin” OR “cyanocobalamine”)—(January 2025).

Web of Science: “phenylketonuria” OR “phenylalanine hydroxylase deficiency” OR “phenylalanine hydroxylase deficient” OR “PKU” OR “hyperphenylalaninemia” OR “BH4 deficiency” OR “BH4 deficient” OR “tetrahydrobiopterin deficiency” OR “tetrahydrobiopterin deficient” OR “PAH deficiency” OR “PAH deficient” OR “phenylketonuric” OR “hyperphenylalaninaemia” (Topic) AND “dietary” OR “supplement” OR “supplementations” OR “supplementation” OR “nutritional” OR “nutrition” OR “diet” OR “diets” OR “vitamin” OR “vitamins” OR “vitaminization” OR “vitaminisation” OR “nutrient” OR “nutrients” OR “micronutrient” OR “micronutrients” OR “water soluble” OR “water-soluble” OR “folic” OR “folate” OR “folacin” OR “pteroylglutamic” OR “pteroyl-L-glutamate” OR “Pteroyl-L-glutamic” OR “cobalamin” OR “cobalamins” OR “cobalamine” OR “cobalamines” OR “cyanocobalamin” OR “cyanocobalamine” (Topic)—(January 2025).

#### 3.1.6. Risk of Bias of Individual Studies

Given that the meta-analysis incorporated non-randomised studies, the risk of bias was evaluated using the Newcastle–Ottawa scale (NOS) [34], a widely used tool for assessing the quality and risk of bias in observational studies such as cohort, case-control, and cross-sectional studies. The adapted NOS version by Modesti et al. [35] was used to assess the cross-sectional studies. The NOS focuses on three main domains:

1. Selection: this domain assesses how well the study defines and selects participants, including the representativeness of the sample and the method of identifying cases and controls;
2. Comparability: this domain evaluates how the study accounts for potential confounding factors by examining the comparability of study groups on important characteristics;
3. Outcome (or exposure): this domain examines the accuracy and reliability of outcome assessment or exposure measurement.

Each study was rated based on the presence and quality of specific criteria within these domains. There was a maximum of 9 points for case-control studies and 10 points for cross-sectional studies, with higher scores indicating a lower risk of bias and better methodological quality. Scores ≥ 7 indicate a low risk of bias, 5–6 reflects a moderate risk, and ≤ 4 suggests a high risk. Each study was independently evaluated by two reviewers (K.B. & M.J.) according to the predefined NOS criteria and any disagreements were resolved through discussion to achieve consensus.

#### 3.1.7. Certainty of Evidence Assessment

The Grading of Recommendations, Assessment, Development, and Evaluation (GRADE) framework [36] was applied by two independent researchers (K.B. & M.J.) to assess the quality of evidence, with any disparities being addressed through discussion. The GRADE approach helps assess the certainty of evidence and the strength of recommendations, enhancing the quality and reliability of systematic review conclusions.

#### 3.1.8. Data Synthesis and Analysis

Statistical analyses were performed using Comprehensive Meta-Analysis Software, version 3.0 (Biostat, Inc., Englewood, CO, USA). A *p*-value < 0.05 was considered statistically significant. The effect size calculations were based on the mean and standard deviation (SD). The vitamin plasma and serum levels, and erythrocyte folate, were used for the effect size measure. The meta-analysis was conducted if at least two identified studies per vitamin were available. Studies that reported data only as a median and range and for which additional details could not be obtained from authors were excluded from the meta-analysis. The original data from the included studies were used for the meta-analysis and the heterogeneity among the studies was evaluated using the Cochran Q statistic (with a *p*-value < 0.1 indicating significant heterogeneity) and the I^2^ statistic. Low heterogeneity was defined as I^2^ < 25%, moderate heterogeneity as I^2^ = 25–50%, and high heterogeneity as I^2^ > 75%. A fixed-effects model was used to aggregate outcomes when the heterogeneity was low (I^2^ < 25%). In cases of substantial heterogeneity (I^2^ > 25%), a random-effects model was employed for the meta-analysis. The effect sizes were presented as standardised mean differences (SMDs) to enable comparison across studies. The SMDs were calculated by dividing the difference between the mean outcome values of the groups by the pooled SD of the outcome values. Forest plots were used to visualise the effect sizes and 95% CIs for each study. Sensitivity analyses were performed by sequentially excluding individual studies and recalculating the pooled estimates to test the robustness of the results. Studies identified as having a high risk of bias were excluded to evaluate their impact on the overall findings. The publication bias was quantitatively evaluated with Begg’s and Egger’s tests. A cumulative meta-analysis was also conducted. Subgroup analysis was conducted to explore potential sources of heterogeneity and assess differences in outcomes across predefined groups. The data were stratified based on the development status of the countries (developed vs. developing) where the publications originated. Comparisons were made between adherent and non-adherent individuals and between individuals with regular and irregular formula intake within each subgroup. For one study [37], the necessary calculations were performed by the researchers following the same methodology applied in the cross-sectional part of the present study. The authors provided data for all patients, and only the data that were relevant to our analysis were collected to ensure they matched the criteria used in our study.

### 3.2. Results

#### 3.2.1. Search Results

**Figure 2:**
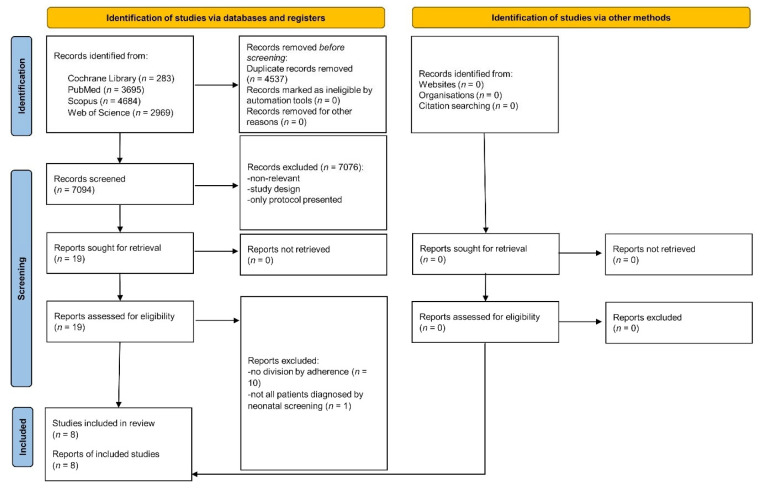
PRISMA 2020 flow diagram.

From the initial 11,631 articles, 4537 duplicates were removed and, after the screening of titles and abstracts, 8 articles met the inclusion criteria (Figure 2).

#### 3.2.2. Study Characteristics

The included studies were published between 2000 [38] and 2025 (present study) and their characteristics are summarised in Table 4. Two studies were conducted in Turkey [22,25], one in Chile [39], one in Switzerland [21], one in Spain [37], one in Greece [20], one in the United Kingdom [38], and one, our study, in Poland. All studies were cross-sectional, with the number of participants ranging from 5 [21] to 69 [37]. One study focused on children [20], one on adolescents [22], three on adults [21,38,39], one included both children and adolescents [25], one was without age restrictions [37], and one, our study, included participants older than 12 years old. All studies included male and female participants. Five studies [20,22,25,37] divided the patients into groups based on their mean Phe concentrations, while three studies [38,39] did so based on formula consumption. One study [32] involved both patients with classical and patients with mild PKU, while two [31,33] did not specify the type of PKU in their populations. The remaining studies exclusively included patients with classical PKU. Table 5 details the dietary characteristics and metabolic status of the participants.

#### 3.2.3. Risk of Bias

Table 6 presents the risk of bias assessment, which indicated that the overall scores for the cross-sectional studies varied from high to moderate, with a range from 0 to 10 points. The low scores reflect the methodological and reporting shortcomings observed in many studies, which undermine the reliability of the reviewed research. Concerning selection bias, most studies focused on specific population groups, so only one study received a point for ‘representativeness of the sample’. Seven studies out of eight (87.5%) employed validated measurement tools to assess vitamin levels, while only one study did not provide details about the tools used. Since blood samples were collected from all participants who were invited to the study, indicating a complete response rate, it can be presumed that there was no non-response among those approached for blood sample collection. As a result, all but one study (87.5%) received a score in this category since the authors did not conduct tests for all patients [38].

Regarding comparability, 75% of the studies controlled at least one main confounding factor and 50% applied additional factors. The evaluation of the outcome, as per NOS criteria, hinged on the assessment of the outcome and the statistical tests that were employed. Points in the first category are granted only if independent blind assessment, record linkage, or self-report methods were implemented. Only one study provided data regarding blinded assessment and thus gained a point. Since the measurement of vitamin levels is objective, it should not impact the results, but points could not be assigned to other studies according to the scale. Complete statistical tests were included in one study. While most studies provided adequate statistical analysis information, they did not present the CIs, which resulted in a deduction in points in this category.

#### 3.2.4. Comparison of Folate Levels in Adherent vs. Non-Adherent PKU Individuals

**Figure 3:**
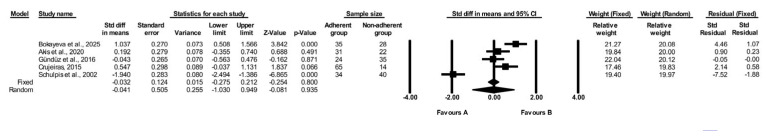
Forest plot of the folate levels in adherent patients (favours B) vs. non-adherent (favours A) (random model). CI—confidence interval; Std—standard; Std diff—standard differences. The analysis includes four published papers [20,22,25,37] and the results of the present study [Bokayeva et al., 2025]. The diamond indicates the overall pooled estimate and its 95% confidence interval.

**Figure 4:**
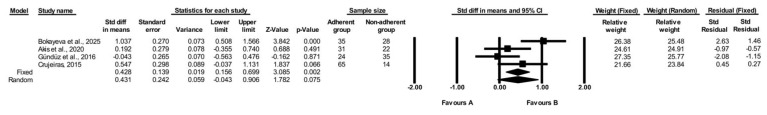
Forest plot of the folate levels in adherent patients (favours B) vs. non-adherent (favours A) without old studies (random model). CI—confidence interval; Std—standard; Std diff—standard differences. The analysis includes three published papers [22,25,37] and the results of the present study [Bokayeva et al., 2025]. The diamond indicates the overall pooled estimate and its 95% confidence interval.

The folate levels were evaluated in five studies [20,22,25,37], including our study (Table 7). To enhance clarity, we included a supplementary table that presents converted folate and vitamin B12 values from the analysed studies in consistent units, facilitating comparison across studies (Table S1). There were no significant differences between the adherent and non-adherent individuals with PKU (random-effects model, SMD: −0.041, 95% CI: −1.030, 0.949, *p* = 0.935, Figure 3). Heterogeneity analysis revealed a high level of variability (Q-value = 65.702, *p* < 0.0001, I^2^ = 93.912%). The sensitivity analysis is presented in Figures S1 and S2 and shows the cumulative analysis findings. After excluding studies with a high risk of bias [20,37], the comparison of folate levels between the adherent and non-adherent PKU groups remained statistically non-significant (random-effects model, SMD: 0.395, 95% CI: −0.253, 1.044, *p* = 0.232, Figure S3). Another analysis was conducted, excluding studies older than 20 years. This analysis comprised four studies [22,25,37], including our own. The comparison of folate levels between adherent and non-adherent PKU individuals using a random-effects model yielded an SMD of 0.431 (random-effects model, 95% CI: −0.043, 0.906, *p* = 0.075, Figure 4). The *p*-value in this case approached statistical significance, suggesting a potential trend. The heterogeneity analysis indicated a substantial degree of variability (Q-value = 9.120, *p* = 0.028, I^2^ = 67.104%).

#### 3.2.5. Comparison of Vitamin B12 Levels in Adherent vs. Non-Adherent PKU Individuals

**Figure 5:**
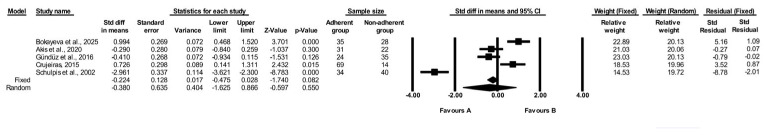
Forest plot of vitamin B12 levels in adherent patients (favours B) vs. non-adherent (favours A) (random model). CI—confidence interval; Std—standard; Std diff—standard differences. The analysis includes four published papers [20,22,25,37] and the results of the present study [Bokayeva et al., 2025]. The diamond indicates the overall pooled estimate and its 95% confidence interval.

**Figure 6:**
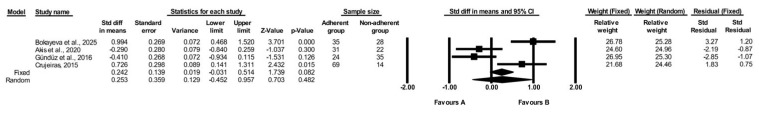
Forest plot of vitamin B12 levels in adherent patients (favours B) vs. non-adherent (favours A) without old studies (random model). CI—confidence interval; Std—standard; Std diff—standard differences. The analysis includes three published papers [22,25,37] and the results of the present study [Bokayeva et al., 2025]. The diamond indicates the overall pooled estimate and its 95% confidence interval.

The meta-analysis of five studies [20,22,25,37], including our study, which evaluated vitamin B12 concentrations revealed no significant difference between the adherent patients with PKU and the non-adherent participants (random-effects model, SMD: −0.380, 95% CI: −1.625, 0.866, *p* = 0.550, Figure 5). The risk of heterogeneity was assessed as high (Q-value = 97.137, *p* < 0.001, I^2^ = 95.882%). The results of the sensitivity analysis are illustrated in Figure S4, while the cumulative analysis findings are presented in Figure S5. The exclusion of studies considered to have a high risk of bias [20,37] did not alter the findings (random-effects model, SMD: 0.395, 95% CI: −0.253, 1.044, *p* = 0.232, Figure S6). The analysis without old studies included four studies [22,25,37], including our own. No significant difference in vitamin B12 levels between adherent and non-adherent individuals with PKU was found (random-effects model, SMD: 0.253, 95% CI: −0.452 to 0.957, *p* = 0.482, Figure 6). Heterogeneity analysis revealed high variability among the studies (Q-value = 20.007, *p* = 0.0002, I^2^ = 85.005%).

#### 3.2.6. Comparison of Folate Levels in Regular Intake vs. Irregular Formula Intake PKU Individuals

One study [21] was excluded from the meta-analysis because the reported folate levels were unusually high, with a standard deviation that was significantly outside the expected range, which raised concerns about potential errors in data reporting.

**Figure 7:**
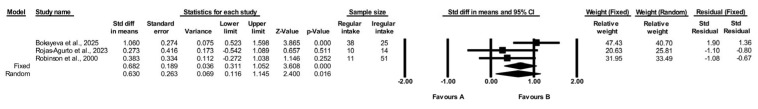
Forest plot of folate levels in regular intake patients (favours B) vs. irregular intake (favours A) (random model). CI—confidence interval; Std—standard; Std diff—standard differences. The analysis includes two published papers [38,39] and the results of the present study [Bokayeva et al., 2025]. The diamond indicates the overall pooled estimate and its 95% confidence interval.

Folate levels were assessed in a total of three studies [38,39], including our own. The analysis comparing individuals with regular and irregular formula intake revealed significant differences (random-effects model, SMD: 0.630, 95% CI: 0.116, 1.145, *p* = 0.016, Figure 7). Heterogeneity analysis demonstrated a moderate degree of variability among the studies (Q-value = 3.666, *p* = 0.160, I^2^ = 45.448%). The sensitivity analysis findings are detailed in Figure S7, and the cumulative analysis is shown in Figure S8.

**Figure 8:**
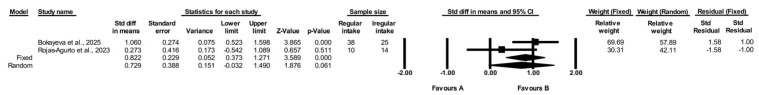
Forest plot of folate levels in regular intake patients (favours B) vs. irregular intake (favours A) without old studies (random model). CI—confidence interval; Std—standard; Std diff—standard differences. The analysis includes one published paper [39] and the results of the present study [Bokayeva et al., 2025]. The diamond indicates the overall pooled estimate and its 95% confidence interval.

A separate analysis was conducted, excluding studies published over 20 years ago. This subset analysis included two studies [39], with our study being one of them. The comparison between irregular and irregular intake groups yielded an SMD of 0.729 (random-effects model, 95% CI: −0.032, 1.490, *p* = 0.061, Figure 8). While not statistically significant, the results suggest a trend toward higher folate levels in the regular intake group, warranting further investigation. The heterogeneity in this analysis was substantial (Q-value = 2.494, *p* = 0.114, I^2^ = 59.905%).

#### 3.2.7. Comparison of Vitamin B12 Levels in Regular Intake vs. Irregular Intake PKU Individuals

**Figure 9:**
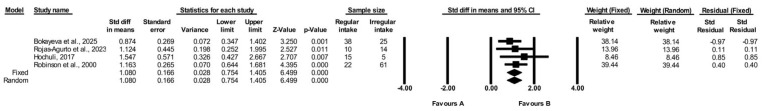
Forest plot of vitamin B12 levels in regular intake patients (favours B) vs. irregular intake (favours A) (random model). CI—confidence interval; Std—standard; Std diff—standard differences. The analysis includes three published papers [21,38,39] and the results of the present study [Bokayeva et al., 2025]. The diamond indicates the overall pooled estimate and its 95% confidence interval.

Vitamin B12 levels were assessed in four studies [21,38,39], including our own. The analysis showed that the vitamin B12 levels were significantly greater in the group with regular intake (fixed-effects model, SMD: 1.080, 95% CI: 0.754, 1.405, *p* < 0.0001, Figure 9). Heterogeneity analysis indicated a low level of variability among the studies (Q-value = 1.360, *p* = 0.715, I^2^ = 0.0%). The sensitivity analysis results are presented in Figure S9, while the cumulative analysis is detailed in Figure S10. Excluding studies identified as having a high risk of bias [21,38] did not change the results (fixed-effects model, SMD: 0.941, 95% CI: 0.490, 1.392, *p* < 0.0001, Figure S11).

**Figure 10:**
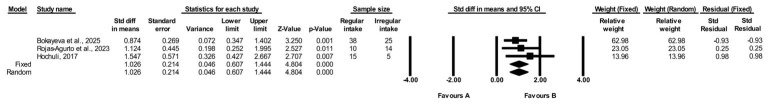
Forest plot of vitamin B12 levels in regular intake patients (favours B) vs. irregular intake (favours A) without old studies (random model). CI—confidence interval; Std—standard; Std diff—standard differences. The analysis includes two published papers [21,39] and the results of the present study [Bokayeva et al., 2025]. The diamond indicates the overall pooled estimate and its 95% confidence interval.

After we excluded studies published more than 20 years ago, the subset included three studies [21,39], including our own. The comparison revealed that the vitamin B12 levels were significantly higher in the regular intake group, with an SMD of 1.026 (fixed-effects model, 95% CI: 0.607 to 1.444, *p* < 0.0001, Figure 10). Heterogeneity within this subset was low (Q-value = 1.198, *p* = 0.549, I^2^ = 0.0%).

#### 3.2.8. Subgroup Analysis

Stratification by region (developed vs. developing countries) revealed no significant differences in folate levels between the adherent and non-adherent individuals in either group (developed: SMD = −0.118, 95% CI: −1.934 to 1.707, *p* = 0.899; developing: SMD = 0.068, 95% CI: −0.308 to 0.445, *p* = 0.722, random-effects model) (Figure S12). Similarly, no significant differences in vitamin B12 levels were observed between the adherent and non-adherent individuals in both the developed and developing countries study subgroups (developed: SMD = −0.407, 95% CI: −2.763 to 1.949, *p* = 0.735; developing: SMD = −0.353, 95% CI: −0.732 to 0.027, *p* = 0.068, random-effects model) (Figure S13).

Stratification by region indicated that the folate levels were significantly higher in individuals with regular formula intake compared to those with irregular intake in developed countries (random-effects model, SMD = 0.749, 95% CI: 0.087 to 1.410, *p* = 0.027), with no significant difference being observed in developing countries (random-effects model, SMD = 0.273, 95% CI: −0.542 to 1.089, *p* = 0.511) (Figure S14).

The individuals with regular formula intake exhibited significantly higher vitamin B12 levels compared to those with irregular intake in both developed countries (fixed-effects model, SMD = 1.073, 95% CI: 0.722 to 1.424, *p* < 0.0001) and developing countries (fixed-effects model, SMD = 1.124, 95% CI: 0.252 to 1.995, *p* = 0.011) (Figure S15), which highlights a consistent pattern across different settings.

#### 3.2.9. Certainty of Evidence Assessment

Table 8 and Table 9 summarise the GRADE certainty assessments for outcomes comparing the vitamin B12 and folate levels in the investigated groups. The analysis is based on cross-sectional studies and reports standardised mean differences to evaluate effect sizes. The publication bias was evaluated using Begg’s and Egger’s tests.

In the assessment between adherent and non-adherent groups (Table 8), the certainty is downgraded for risk of bias, inconsistency, and imprecision issues. Residual confounding factors are likely to underestimate the true effect, which supports increased confidence in the findings. However, the high inconsistency and imprecision reduce the certainty, which suggests the need for more robust study designs. A high inconsistency reflects substantial heterogeneity among studies. Imprecision is addressed based on the wide confidence intervals and relatively small sample sizes in several of the included studies. Indirectness was not identified as a concern in this analysis and the included studies directly addressed the research question, comparing the vitamin B12 and folate levels in adherent and non-adherent individuals with PKU.

In the assessment between the regular and irregular formula intake groups (Table 9), the certainty was downgraded due to risk of bias, imprecision issues, and, to a lesser extent, moderate inconsistency for folate outcomes. For vitamin B12, the certainty is higher due to the consistency of the findings across studies, with minimal heterogeneity being observed, which reflects robust agreement in the direction and magnitude of effects. Conversely, for folate, moderate heterogeneity and a wide CI suggest that the results may vary depending on the study parameters. While the overall certainty for vitamin B12 was rated as low, the folate findings remain very low, which underscores the need for higher-quality studies with adequate sample sizes to confirm the observed trends.

## 4. Discussion

Our cross-sectional study explored the relationship between adherence to the PKU diet and the levels of vitamin B12 and folate in individuals with PKU. Adherent individuals, as defined by Phe levels, had significantly higher levels of vitamin B12 and folate than non-adherent individuals. Similarly, participants with regular formula intake showed significantly higher levels of these nutrients compared to those with irregular intake. However, the meta-analysis revealed that the folate and vitamin B12 levels did not differ significantly between the adherent and non-adherent individuals with PKU across studies. In contrast, individuals with regular formula intake exhibited significantly higher vitamin B12 (*p* < 0.0001) and folate (*p* < 0.016) levels compared to those with irregular intake.

Regular formula intake emerged as a critical factor in maintaining higher vitamin B12 levels in the regular intake group compared to the irregular intake group. Adherence to the Phe-free mixture appears to be the primary factor that contributed to the higher vitamin B12 levels observed in the adherent group compared to the non-adherent (defined based on Phe levels) group in the cross-sectional part of the present study. The Phe-free formula is specifically fortified with essential vitamins, including vitamin B12, and regular consumption ensured sufficient intake among adherent and regular formula intake individuals. Non-adherent individuals with irregular formula intake have the potential to meet their vitamin B12 requirements through high-protein foods such as meat, fish, eggs, and dairy products [40,41]. However, their actual intake may vary, as some may rely more on carbohydrate-rich foods like bread, biscuits, and pasta rather than vitamin B12-rich animal products. This dietary pattern could contribute to lower vitamin B12 levels in non-adherent individuals (in fact with irregular formula intake), which may explain why a deficiency was observed in one patient who belonged to both groups.

Previous studies have reported low vitamin B12 levels in some patients with PKU [18,20,42,43] and other studies have assumed that patients with PKU who have discontinued dietary therapy or are following it partially may face an increased risk of nutritional deficiencies [38,44,45]. Kose et al. [18] observed that strict adherence to the diet was linked to higher serum vitamin B12 levels. Among the adherent individuals in our study, 12 (34.3%) had elevated vitamin B12 levels, whereas only two patients (7.1%) in the non-adherent group exhibited high vitamin B12 concentrations. Similarly, 11 (29%) individuals in the regular intake group had elevated vitamin B12 levels compared to 3 (12%) in the irregular group. Our findings highlight the variability in vitamin B12 levels among patients with PKU, emphasising that formula intake is the primary determinant of this vitamin status. Strict dietary adherence to recommended formula intake supports adequate levels, and monitoring is crucial to prevent deficiencies as well as consider elevated concentrations. Generally, higher vitamin B12 doses are believed to be safe since any excess is expected to be excreted with urine. However, very high doses, such as those applied to treat a deficiency, may result in some side effects [46].

No folate deficiency was found and the high folate levels that were observed in the vast majority of patients may be attributed to the widespread availability of folate in various food sources. Folate is naturally found in dark green leafy vegetables, fruits and fruit juices, nuts, beans, peas, seafood, eggs, dairy products, meat, poultry, and grains [47,48]. The PKU diet, primarily composed of vegetables and fruits, with a total of up to five portions and without the need for measurement as they contain less than 75 mg of Phe per 100 g [3], is additionally supplemented with Phe-free amino acid formulas that are typically fortified with essential vitamins and minerals including folic acid [49]. Studies have linked the elevated folate levels in individuals with PKU to the substantial inclusion of vegetables in the Phe-restricted diet [38,50,51]. Irregular formula intake individuals (including non-adherent individuals) may still maintain adequate folate levels through the diverse range of folate-rich foods. Interestingly, 42.9% of adherent individuals and 17.9% of non-adherent individuals had folate levels above the normal range, while high folate levels were observed in 44.7% of the regular formula intake group compared to 12.0% of the irregular intake group. Another Polish study investigating the plasma folic acid concentrations in children with PKU found that 75.3% of the 73 patients had serum folic acid concentrations above the upper reference level [52]. Stølen et al. [17] documented that 94% of paediatric and 73% of adult Norwegian patients with PKU had a folic acid intake above the recommended daily intake. More importantly, 91% of children and 73% of adults had plasma folate levels above the upper reference level, with none exhibiting low plasma folate levels. The authors attributed their findings to the high folic acid content in protein substitutes. Elevated folate concentrations have also been reported in other studies [16,45,53]. The issue of high folate concentrations due to the elevated folate content in protein substitutes has been discussed in previous reviews [14,54]. Maintaining an appropriate balance of protein, energy, and nutrients in the PKU diet can be challenging, as protein substitutes are prescribed based on individual Phe tolerance and body weight. Consequently, folate intake is influenced by both the dosage of protein substitutes and the total protein equivalent, which is derived from a combination of natural protein sources and protein substitutes. According to a recent review [55], some authors have raised concerns about the potential effects of excessive folate intake, but there is currently no conclusive evidence to suggest that high folate consumption leads to adverse effects.

Overall, the findings highlight that adherence to the PKU diet (including consumption of amino acid mixture) provides an adequate and reliable source of vitamin B12 and folate, which emphasises the effectiveness of the PKU diet in meeting the nutritional needs of adherent individuals while ensuring metabolic control. Participants in the cross-sectional study who adhered to the diet or regularly consumed formula exhibited significantly higher vitamin B12 and folate levels compared to their non-adherent or irregular intake counterparts (what could, at least in part, be explained by overlapping groups). The results of the meta-analysis suggest that the intake of the formula is the critical point, positively influencing individuals’ vitamin B12 and folate status. Maintaining targeted Phe levels may not necessarily be related to adequate formula intake.

To our knowledge, no prior meta-analysis has systematically compared the vitamin B12 and folate levels in adherent versus non-adherent individuals with PKU or in those with regular versus irregular formula intake. Therefore, the present study represents the first attempt to synthesise the available evidence on this topic. The meta-analysis revealed no significant differences in vitamin B12 and folate levels between adherent and non-adherent PKU individuals (as defined by Phe levels) across studies. When older studies were excluded, the comparison of folate levels between adherent and non-adherent PKU individuals revealed a trend toward higher folate levels in the adherent group, although this difference did not reach statistical significance (random-effects model, SMD: 0.431, 95% CI: −0.043, 0.906, *p* = 0.075). Similarly, the vitamin B12 levels remained non-significantly different between the adherent and non-adherent groups in the subset of more recent studies. In contrast, comparisons between regular and irregular formula intake groups consistently showed significantly higher folate and vitamin B12 levels in individuals with regular intake, reinforcing the critical role of formula consumption in maintaining adequate micronutrient status. Notably, the vitamin B12 differences remained statistically significant even after the older studies were excluded, which supports a strong and consistent association, while the folate levels showed a non-significant result that was close to significance but did not quite reach it (random-effects model, SMD: 0.729, 95% CI: −0.032, 1.490, *p* = 0.061). Our subgroup analyses further revealed that the positive association between regular formula intake and micronutrient status was more pronounced in developed countries, particularly for folate levels, which may reflect differences in dietary management and diet. Vitamin B12 levels, however, were consistently higher in regular intake groups across both developed and developing countries, which emphasized the critical role of formula in maintaining adequate vitamin B12 status in patients with PKU globally.

Interestingly, Hochuli et al. [21] reported that, although less adherent adult patients consume more natural protein, it does not fully compensate for the reduced vitamin B12 supply from formula. They supposed that their results reflected real-life dietary behaviours in adult patients with PKU, where many relax their dietary restrictions, particularly regarding natural protein and formula intake, while still maintaining some level of dietary control. However, this reduced adherence puts patients at risk for nutritional deficiencies and imbalances, which leads to an increased intake of saturated fats and insufficient supply of protein, vitamin B12, and selenium compared to more adherent patients. Rojas-Agurto et al. [39] compared patients with PKU who continued protein substitute intake beyond age 18 with those who discontinued it. The second group, following a predominantly vegan diet with limited essential amino acids, exhibited significantly lower blood vitamin B12 levels, which underscored the importance of a formula-based diet for maintaining adequate nutrient concentrations.

A key strength of our study is the integration of original data from a cross-sectional study with a systematic review and meta-analysis. The adequate sample size in our original study, along with the rigorous classifications for both adherence status and formula intake regularity, enhances the reliability of our findings. The inclusion and exclusion criteria for the original study were carefully designed, with a focus on individuals with classic PKU under continuous treatment and minimising confounding factors. However, there are some limitations. We relied solely on serum B12 levels as a biomarker without considering holo-transcobalamin or methylmalonic acid levels, additional markers for assessing B12 status. Moreover, our study included a mixed population of adults and children, which could introduce variability in the results due to age-related differences. The methods used in the analysed studies to assign individuals to adherent and non-adherent groups may have significantly influenced the results that were obtained. For example, Rojas-Agurto [31] categorised patients into two groups: PKU-1 included participants who continued nutritional treatment with adequate Phe-free protein substitutes and maintained strict follow-up, while PKU-2 included those who discontinued protein substitutes at age 18. Gunduz [30] divided patients into well-controlled and poorly controlled subgroups based on their mean serum phenylalanine levels from the past year. Robinson’s study [38] categorised patients into strict—on a low-Phe diet with supplements, and relaxed—consuming ~1 g/kg/day of protein, half from natural sources and half from supplements. Additionally, regarding the definition of adherence, while plasma Phe levels are a useful measure of adherence, they may not fully reflect formula intake, which is essential for ensuring adequate phenylalanine-free protein and nutrient supply. To the best of our knowledge, this is the first meta-analysis to systematically compare the vitamin B12 and folate levels between not only adherent and non-adherent individuals with PKU but also between those with regular and irregular formula intake. This dual approach represents a significant strength, as it captures distinct but related aspects of nutritional management in PKU. We aimed to create a more homogeneous and well-defined population by applying strict inclusion criteria and focusing exclusively on individuals who were diagnosed through neonatal screening. The data were also stratified by the development status of the countries to identify potential factors that may influence our findings. While some analyses—particularly those comparing vitamin B12 levels between regular and irregular formula intake groups—demonstrated low heterogeneity, others, especially those involving adherence status, showed high heterogeneity. Moreover, the relatively small number of studies included in several analyses may limit the statistical power and broader applicability of the findings. This variability calls for caution when interpreting and generalising the results. While we attempted to minimise the heterogeneity through sensitivity analyses and subgroup stratifications, the inability to comprehensively evaluate all contributing factors due to incomplete or inconsistent reporting in the included studies remains a limitation of the current meta-analysis. This highlights the need for future studies to provide more standardised and detailed reporting in order to facilitate the deeper exploration of sources of heterogeneity.

An evaluation of potential risks of bias in our meta-analysis revealed some concerns that should be considered when interpreting the findings. Selection bias was linked to the representativeness and sample size. In the outcome evaluation category, only one study received a point since the other authors did not provide information about the blinding process during measurement, even though vitamin level measurements are inherently objective. Additionally, the lack of confidence intervals led to lower scores in the statistical domain, although other aspects of the statistical analyses were adequate. The NOS assessment tool has limitations, as it relies on subjective judgment and focuses mainly on study design, which may overlook other biases, such as selective outcome reporting or conflicts of interest. Furthermore, the NOS does not provide a quantitative measure of bias magnitude. Lastly, publication bias was assessed, but a reliable assessment was not possible due to the small number of studies. These limitations highlight the need for cautious interpretation of our findings.

Our findings could be influenced by different factors. Research in the field of PKU is limited due to the relatively small patient population (in comparison to common diseases). Variations in vitamin status among study participants may be linked to regional diet patterns. Additionally, shifts in treatment strategies or changes in the composition of formulas (e.g., more recently, formulas have a higher vitamin B12 content) could impact the outcomes that were observed. The patient demographics, including age, sex, and ethnicity, also varied across the included studies, which may have introduced heterogeneity in the results. The method of assigning patients to the adherent and non-adherent groups may have influenced the results. Our classification method may have contributed to variability in the observed vitamin B12 and folate statuses, as plasma Phe levels reflect dietary adherence but may also be influenced by individual metabolic differences and other factors, such as the type of PKU. The inclusion of studies with unspecified or mixed PKU types may have added to the variability, as not all studies exclusively focused on patients with classical PKU. One study included patients with both classical and mild PKU [21], while two studies [20,39] did not specify the type of PKU.

Meta-regression, network meta-analysis, and subgroup analysis based on the sex, type of protein substitute, and metabolic control of the participants were not feasible. Subgroups had a limited number of studies, which may have influenced the results. The results of the meta-analysis should be interpreted with caution due to the low quality of evidence as assessed by GRADE and all study limitations. Larger studies are needed to confirm our findings.

## 5. Conclusions

The cross-sectional data demonstrated significantly higher levels of folate and vitamin B12 among adherent individuals (as defined by Phe levels) and those with consistent formula consumption. The meta-analysis showed no significant differences in vitamin B12 and folate levels between the adherent and non-adherent groups across studies. In contrast, regular formula intake was consistently associated with significantly higher vitamin B12 levels and a trend toward elevated folate levels, which emphasised the crucial role of formula in maintaining nutrient status, the primary determinant of vitamin B12 levels in patients with PKU. Future research with larger, standardised cohorts is needed to clarify the complex interactions between adherence, formula intake, and nutrient status.

## Figures and Tables

**Table 1:** Folate reference ranges by age and sex.

Age Group	Females (ng/mL)	Males (ng/mL)
12–13 years	1–10	1.5–11
13–18 years	1.2–7.1	1.2–8.8
18–20 years	3.8–16	
>20 years	3.9–26.8	

**Table 2:** Demographic, anthropometric, metabolic, and biochemical parameters of the adherent and non-adherent PKU groups.

Parameter	Adherent PKU Group Median (Q1–Q3); Mean ± SD	Non-Adherent PKU Group Median (Q1–Q3); Mean ± SD	*p*-Value
*n*	35	28	
Age (years)	19.00 (14.05–28.25); 20.88 ± 7.33	22.75 (17.00–29.78); 24.01 ± 8.38	0.109 ^1^
Sex (*n* (%))			
Female	23 (65.7%)	14 (50.0%)	0.208 ^2^
Male	12 (34.3%)	14 (50.0%)	
BMI (kg/m^2^) BMI-IOTF corrected (kg/m^2^)	21.90 (19.82–24.09); 21.71 ± 3.35 23.46 (20.18–24.77); 22.68 ± 3.69	23.25 (20.62–29.65); 24.78 ± 5.42 23.56 (21.09–29.78); 25.11 ± 5.22	0.067 ^1^ 0.142 ^1^
Phe mean (mg/dL)	7.62 (5.79–8.93); 7.09 ± 2.13	15.42 (12.92–17.88); 15.62 ± 3.53	<0.0001 ^3^*
Phe median (mg/dL)	6.80 (5.22–8.66); 6.72 ± 2.30	15.09 (12.46–17.62); 15.47 ± 3.48	<0.0001 ^3^*
Abnormal values (%)	23.7 (4.5–33.3); 20.9 ± 15.1	100 (79.0–100); 88.9 ± 14.9	0.0001 ^1^*
Vitamin B12 (pg/mL)	732.0 (576.5–919.0); 767.6 ± 264.5 95% CI (676.78–858.52)	491.5 (369.2–675.8); 524.7 ± 216.4 95% CI (440.76–608.59)	<0.001 ^4^*
Folate (ng/mL)	13.40 (12.35–14.60); 13.44 ± 1.96 95% CI (12.80–14.12)	10.05 (7.97–12.90); 10.62 ± 3.36 95% CI (9.32–11.93)	<0.001 ^3^*

*—statistically significant; ^1^—Mann–Whitney U-test; ^2^—Chi-square test, ^3^—Welch’s *t*-test; ^4^—Student’s *t*-test; significance level—0.05; BMI—body mass index, CI—confidence interval, IOTF—International Obesity Task Force, SD—standard deviation.

**Table 3:** Demographic, anthropometric, metabolic, and biochemical parameters of patients with PKU with regular and irregular intakes of formula.

Parameter	Regular PKU Group Median (Q1–Q3); Mean ± SD	Irregular PKU Group Median (Q1–Q3); Mean ± SD	*p*-Value
*n*	38	25	
Age (years)	19.05 (14.1–27.4); 20.82 ± 7.25	24.2 (17–29.6); 24.48 ± 8.49	0.087 ^1^
Sex (*n* (%))			
Female	25 (65.8%)	12 (52.0%)	0.161 ^2^
Male	13 (34.2%)	13 (48.0%)	
BMI (kg/m^2^) BMI-IOTF corrected (kg/m^2^)	21.65 (19.81–23.55); 21.80 ± 3.60 23.47 (21.24–24.78); 23.39 ± 4.15	24.35 (21–29.65); 25.01 ± 5.35 25.21 (21.75–28.88); 25.94 ± 5.39	0.012 ^3^* 0.222 ^4^
Phe mean (mg/dL)	8.17 (6.02–9.59); 8.16 ± 3.17	16.66 (11.65–18.7); 15.02 ± 4.76	<0.0001 ^3^*
Phe median (mg/dL)	7.86 (5.93–9.78); 7.92 ± 3.28	15.91 (11.36–17.73); 14.70 ± 5.04	<0.0001 ^3^*
Abnormal values (%)	32.3 (6.1–40.1); 32.7 ± 30.2	100 (66.7–100); 79.0 ± 28.7	0.0001 ^1^*
Vitamin B12 (pg/mL)	721.5 (613.5–905); 746.7 ± 228.4 95% CI (671.61–821.76)	449 (358–548); 527.4 ± 281.9 95% CI (411.05–643.75)	0.0001 ^1^*
Folate (ng/mL)	13.45 (12.4–14.6); 13.32 ± 2.25 95% CI (12.58–14.06)	10.1 (8.1–12.7); 10.48 ± 3.23 95% CI (9.15–11.81)	<0.001 ^3^*

*—statistically significant; ^1^—Mann–Whitney U-test; ^2^—Chi-square test, ^3^—Welch’s *t*-test; ^4^—Student’s *t*-test; significance level—0.05; BMI—body mass index, CI—confidence interval, IOTF—International Obesity Task Force, SD—standard deviation.

**Table 4:** Characteristics of included studies and studied individuals.

Author	Year	Country (Region)	Groups	*n* Included	*n* Completed	Age [Years] ^1^	BMI [kg/m^2^] ^1^	Sex [% of Women]
Bokayeva et al.	2025	Poland	Adherent	35	35	20.9 ± 7.3	21.7 ± 3.4	65.7
			Non-adherent	28	28	24.0 ± 8.4	24.8 ± 5.4	50
			Regular	38	38	20.82 ± 7.25	21.80 ± 3.60	65.8%
			Irregular	25	25	24.48 ± 8.49	25.01 ± 5.35	52.0%
Rojas-Agurto et al. [39]	2023	Chile	Regular ^2^	10	10	23.5 (19–26) ^4^	24.3 (22.4–28.5) ^4^	50
			Irregular ^3^	14	14	22.5 (18.5–25.5) ^4^	26.7 (24–29.9) ^4^	36
Akış et al. [25]	2020	Turkey	Adherent ^5^	31	31	9.5 (5.0–18.0) ^7^	NI	38
			Non-adherent ^6^	22	22			
Hochuli et al. [21]	2017	Switzerland	Regular ^8^	15	15	32.0 ± 12.0	24.6 ± 4.3	53
			Irregular ^9^	5	5	39.0 ± 8.4	20.6 ± 2.1	20
Gündüz et al. [22]	2016	Turkey	Adherent ^10^	24	24	13.1 ± 2.4	19.1 ± 2.1	33
			Non-adherent ^11^	35	35	14.1 ± 2.9	18.9 ± 1.9	54
Crujeiras et al. [37]	2015	Spain	Adherent	69	69 ^12^	10.9 (1–92) ^13^	NI	47.8% 50%
			Non-adherent	14	14	23.5 (3–30) ^13^		
Schulpis et al. [20]	2002	Greece	Adherent ^10^	34	34	6.8 ± 1.5	NI	NI
			Non-adherent ^11^	40	40	8.0 ± 3.2		
Robinson et al. [38]	2000	The United Kingdom	Regular ^14^	22	22 ^16^	24.0 ^18^	NI	NI
			Irregular ^15^	61	61 ^17^	21.0 ^18^		

^1^—mean ± standard deviation; ^2^—patients under diet treatment; ^3^—patients who discontinued the protein substitution at 18 years of age; ^4^—median (25th–75th centile); ^5^—patients with high adherence; ^6^—patients with low adherence; ^7^—median (min–max); ^8^—regular AAM intake; ^9^—AAM intake below the prescribed amount; ^10^—well-controlled; ^11^—poorly controlled; ^12^—folate concentration analysis was performed in 65 participants; ^13^—mean (min–max); ^14^—strict diet; ^15^—combined data from relaxed diet group and unrestricted group; ^16^—folate concentration analysis was performed in 11 participants; ^17^—folate concentration analysis was performed in 51 participants; ^18^—median. BMI—body mass index; NI—no information.

**Table 5:** Characteristics of diet and metabolic status of studied individuals.

Author	Year	Groups	Phe Intake [mg/d]	Mean/Median Phe Levels ^1^	Medical Control	Last Phe Level [μmol/L] ^1^	Last Tyr Level [μmol/L] ^1^
Bokayeva et al.	2025	Adherent	NI	7.09 ± 2.13 mg/dL	Yes	NI	NI
		Non-adherent		15.62 ± 3.53 mg/dL	Yes		
		Regular	NI	8.16 ± 3.17 mg/dL	Yes	NI	NI
		Irregular		15.02 ± 4.76 mg/dL	Yes		
Rojas-Agurto et al. [39]	2023	Regular ^2^	600 (400–800) ^4^	NI	Yes	260.3 (170.0–642.0) ^4^	46.60 (33.1–49.7) ^4^
		Irregular ^3^	1200 (500–1700) ^4^		No	781.0 (636.0–1035.1) ^4^	35.90 (33.1–55.2) ^4^
Akış et al. [25]	2020	Adherent ^5^	NI	NI	Yes	357.19 (121.10–514.60) ^7^	NI
		Non-adherent ^6^			Yes	696.21 (441.90–1035.20) ^7^	
Hochuli et al. [21]	2017	Regular ^8^	NI	NI	NI	650 ± 283	NI
		Irregular ^9^				760 ± 350	
Gündüz et al. [22]	2016	Adherent ^10^	300–900 ^12^	NI	NI	306.1 ± 78.0	NI
		Non-adherent ^11^				720.8 ± 196.7	
Crujeiras et al. [37]	2015	Adherent	NI	276.6 ± 133.4 μmol/L (4.57 ± 2.20 mg/dL)	NI	NI	NI
		Non-adherent		867.1 ± 273.7 μmol/L (14.32 ± 4.52 mg/dL)			
Schulpis et al. [20]	2002	Adherent ^10^	NI	NI	NI	192 ± 115	NI
		Non-adherent ^11^				599 ± 16	
Robinson et al. [38]	2000	Regular ^13^	NI	NI	Yes	NI	NI
		Irregular ^14^			Yes		

^1^—mean ± standard deviation; ^2^—patients under diet treatment; ^3^—patients who discontinued the protein substitution at 18 years of age; ^4^—median (25th–75th centile); ^5^—patients with high adherence; ^6^—patients with low adherence; ^7^—median (min–max); ^8^—regular AAM intake; ^9^—AAM intake below the prescribed amount; ^10^—well-controlled; ^11^—poorly controlled; ^12^—range; ^13^—strict diet; ^14^—combined data from relaxed diet group and unrestricted group. Phe—phenylalanine; Tyr—tyrosine; NI—no information.

**Table 6:** Newcastle–Ottawa quality assessment scale.

Study (First Author)	Selection				Comparability	Outcome		Overall Score
	Representativeness of the Sample	Sample Size	Non- Respondents	Ascertainment of Exposure	Based on Design and Analysis	Assessment of Outcome	Statistical Test	
Bokayeva et al., 2025	+	+	+	+ +	+ +	++	+	10
Rojas-Agurto et al. [39], 2023			+	+ +	+ +			5
Akış et al. [25], 2020			+	+ +	+ +			5
Hochuli et al. [21], 2017			+	+ +	+			4
Gündüz et al. [22], 2016			+	+ +	+ +			5
Crujeiras et al. [37], 2015			+	+ +				3
Schulpis et al. [20], 2002			+	+ +				3
Robinson et al. [38], 2000								0

+—1 point awarded.

**Table 7:** Comparison of vitamin status in studied individuals.

Author	Year	Groups	Folate ^1^	Vitamin B12 ^1^
Bokayeva et al.	2025	Adherent	13.4 ± 1.96 ^2,3^	767.6 ± 264.5 ^2,4^
		Non-adherent	10.63 ± 3.36 ^2,3^	524.7 ± 216.4 ^2,4^
		Regular	13.32 ± 2.25 ^2,3^	746.7 ± 228.4 ^2,4^
		Irregular	10.48 ± 3.23 ^2,3^	527.4 ± 281.9 ^2,4^
Rojas-Agurto et al. [39]	2023	Regular ^5^	25.69 ± 7.58 ^2,3,7,8^	706.4 ± 330.4 ^2,8,9^
		Irregular ^6^	23.68 ± 7.19 ^2,3,7,8^	383.4 ± 253.2 ^2,8,9^
Akış et al. [25]	2020	Adherent ^10^	37.2 ± 9.5 ^2,12^	282.2 ± 128.5 ^2,13^
		Non-Adherent ^11^	35.3 ± 10.4 ^2,12^	318.9 ± 123.2 ^2,13^
Hochuli et al. [21]	2017	Regular ^14^	98 ± 290 ^2,7,16^	540 ± 208 ^2,9^
		Irregular ^15^	14 ± 3 ^2,7,16^	251 ± 75 ^2,9^
Gündüz et al. [22]	2016	Adherent ^17^	32.4 ± 9.7 ^2,7,12^	256.5 ± 139.3 ^2,13^
		Non-Adherent ^18^	32.8 ± 9.0 ^2,7,12^	308.8 ± 119.1 ^2,13^
Crujeiras et al. [37]	2015	Adherent	19.8 ± 7.0 ^3,7,19^	749.6 ± 331.7 ^4,19^
		Non-adherent	15.8 ± 8.6 ^3,7,19^	515.9 ± 264.5 ^4,19^
Schulpis et al. [20]	2002	Adherent ^17^	2.35 ± 1.3 ^2,12^	98.5 ± 22.3 ^2,13^
		Non-adherent ^18^	5.8 ± 2.1 ^2,12^	240.8 ± 62 ^2,13^
Robinson et al. [38]	2000	Regular ^20^	476 ± 258 ^16,22^	468.7 ± 199.7 ^9,23^
		Irregular ^21^	399.8 ± 184.8 ^16,22^	303.6 ± 115.2 ^9,23^

^1^—mean ± standard deviation; ^2^—serum; ^3^—ng/mL; ^4^—pg/mL; ^5^—patients under diet treatment; ^6^—patients who discontinued the protein substitution at 18 years of age; ^7^—folic acid; ^8^—data were received from authors; ^9^—ng/L; ^10^—patients with high adherence; ^11^—patients with low adherence; ^12^—nmol/L; ^13^—pmol/L; ^14^— regular AAM intake; ^15^—AAM intake below the prescribed amount; ^16^—µg/L; ^17^—well-controlled; ^18^—poorly controlled; ^19^—plasma; ^20^—strict diet; ^21^—combined data from relaxed diet group and unrestricted group; ^22^—red blood cells folate; ^23^—no info about measurement material.

**Table 8:** Certainty of evidence assessment for vitamin B12 and folate in adherent vs. non-adherent individuals with PKU.

Certainty Assessment							No. of Patients		Effect	Certainty
Outcome and No. of Studies	Study Design	Risk of Bias	Inconsistency	Indirectness	Imprecision	Other Considerations	Adherence	Non-Adherence	Absolute (95% CI)	
Vitamin B12—5	non-randomised studies	serious ^a^	serious ^b^	not serious	serious ^c^	all plausible residual confounding would reduce the demonstrated effect	189	139	SMD 0.38 SD lower (1.625 lower to 0.866 higher)	⨁◯◯◯ Very low ^a,b,c^
Folate—5	non-randomised studies	serious ^a^	serious ^d^	not serious	serious ^c^	all plausible residual confounding would reduce the demonstrated effect	189	139	SMD 0.041 SD lower (1.03 lower to 0.949 higher))	⨁◯◯◯ Very low ^a,c,d^

^a^—downgraded by one level due to lack of control for confounding variables and small sample sizes in some studies; ^b^—large heterogeneity (Q-value = 97.137, *p* < 0.001, I^2^ = 95.882%). Variations in vitamin status among participants may reflect regional dietary patterns, treatment strategies, formula composition, and differences in demographics such as age, sex, and ethnicity; ^c^—downgraded by one level since confidence intervals were wide, including clinically irrelevant and significant thresholds, reflecting imprecision due to small sample size; ^d^—large heterogeneity (Q-value = 65.702, *p* < 0.0001, I^2^ = 93.912%). Variations in vitamin status among participants may reflect regional dietary patterns, treatment strategies, formula composition, and differences in demographics such as age, sex, and ethnicity. CI—confidence interval; SMD—standardised mean difference.

**Table 9:** Certainty of evidence assessment for vitamin B12 and folate in regular vs. irregular individuals with PKU.

Certainty Assessment							No. of Patients		Effect	Certainty
Outcome and No. of Studies	Study Design	Risk of Bias	Inconsistency	Indirectness	Imprecision	Other Considerations	Adherence	Non-Adherence	Absolute (95% CI)	
Vitamin B12—4	non-randomised studies	serious ^a^	not serious ^b^	not serious	not serious	all plausible residual confounding would reduce the demonstrated effect	85	105	SMD 1.08 SD higher (0.754 higher to 1.405 higher)	⨁⨁◯◯ Low ^a,b,c^
Folate—3	non-randomised studies	serious ^c^	serious ^d^	not serious	serious ^e^	all plausible residual confounding would reduce the demonstrated effect	59	90	SMD 0.63 SD higher (0.116 higher to 1.145 higher)	⨁◯◯◯ Very low ^a,c,d^

^a^—downgrade by one level for risk of bias, as the inclusion of a very high-risk study [38] and small sample sizes introduces some uncertainty, even though sensitivity analysis and analysis excluding high-risk-of-bias studies provides reassurance; ^b^—heterogeneity is minimal (Q = 1.360, *p* = 0.715, I^2^ = 0.0%), indicating that the results are consistent across studies. The direction and magnitude of the effect size are similar among studies; ^c^—downgraded by one level due to high risk of bias in one included study [38] and small sample sizes. Sensitivity analysis showed that removing this study rendered the results non-significant (although close to significance), highlighting its influence on the overall findings; ^d^—moderate heterogeneity (Q-value = 3.666, *p* = 0.160, I^2^ = 45.448%); ^e^—downgraded by one level. The confidence interval is wide, spanning from a small to a large effect size, reflecting uncertainty in the true magnitude of the effect. CI—confidence interval; SMD—standardised mean difference.

## Data Availability

Data are available from the corresponding author upon reasonable request.

## Supplementary Materials

The following supporting information can be downloaded at: https://www.mdpi.com/article/10.3390/metabo15070438/s1, Table S1. Comparison of vitamin status in studied individuals (with converted units); Figure S1. Sensitivity analysis by the jack-knife approach presenting mean differences with 95% confidence interval in folate levels between adherent patients (favours B) vs. non-adherent patients (favours A) (random model). CI—confidence interval; Std diff—standard differences. The analysis includes four published papers [20,22,25,37] and the results of the present study [Bokayeva et al., 2025]; Figure S2. Cumulative meta-analysis of the folate levels in adherent patients (favours B) vs. non-adherent patients (favours A). CI—confidence interval; Std—standard; Std diff—standard differences. The analysis includes four published papers [20,22,25,37] and the results of the present study [Bokayeva et al., 2025]; Figure S3. Sensitivity analysis presenting mean differences with a 95% confidence interval in folate levels between adherent patients (favours B) vs. non-adherent patients (favours A) (random model) after exclusion of studies with an overall high risk of bias. CI—confidence interval; Std—standard; Std diff—standard differences. The analysis includes two published papers [22,25] and the results of the present study [Bokayeva et al., 2025]; Figure S4. Sensitivity analysis by the jack-knife approach presenting mean differences with a 95% confidence interval in vitamin B12 levels between adherent patients (favours B) vs. non-adherent patients (favours A) (random model). CI—confidence interval; Std diff—standard differences. The analysis includes four published papers [20,22,25,37] and the results of the present study [Bokayeva et al., 2025]; Figure S5. Cumulative meta-analysis of the vitamin B12 levels in adherent patients (favours B) vs. non-adherent patients (favours A). CI—confidence interval; Std—standard; Std diff—standard differences. The analysis includes four published papers [20,22,25,37] and the results of the present study [Bokayeva et al., 2025]; Figure S6. Sensitivity analysis presenting mean differences with a 95% confidence interval in vitamin B12 levels between adherent patients (favours B) vs. non-adherent patients (favours A) (random model) after exclusion of studies with an overall high risk of bias. CI—confidence interval; Std—standard; Std diff—standard differences. The analysis includes two published papers [22,25] and the results of the present study [Bokayeva et al., 2025]; Figure S7. Sensitivity analysis by the jack-knife approach presenting mean differences with 95% confidence interval in folate levels between regular patients (favours B) vs. irregular patients (favours A) (random model). CI—confidence interval; Std diff—standard differences. The analysis includes two published papers [38,39] and the results of the present study [Bokayeva et al., 2025]; Figure S8. Cumulative meta-analysis of the folate levels in regular patients (favours B) vs. irregular patients (favours A). CI—confidence interval; Std—standard; Std diff—standard differences. The analysis includes two published papers [38,39] and the results of the present study [Bokayeva et al., 2025]; Figure S9. Sensitivity analysis by the jack-knife approach presenting mean differences with a 95% confidence interval in vitamin B12 levels between regular patients (favours B) vs. irregular patients (favours A) (random model). CI—confidence interval; Std diff—standard differences. The analysis includes three published papers [21,38,39] and the results of the present study [Bokayeva et al., 2025]; Figure S10. Cumulative meta-analysis of the vitamin B12 levels in regular patients (favours B) vs. irregular patients (favours A). CI—confidence interval; Std—standard; Std diff—standard differences. The analysis includes three published papers [21,38,39] and the results of the present study [Bokayeva et al., 2025]; Figure S11. Sensitivity analysis presenting mean differences with a 95% confidence interval in vitamin B12 levels between regular patients (favours B) vs. irregular patients (favours A) (random model) after exclusion of studies with an overall high risk of bias. CI—confidence interval; Std—standard; Std diff—standard differences. The analysis includes 1 published paper [39] and the results of the present study [Bokayeva et al., 2025]; Figure S12: Subgroup meta-analysis of folate levels according to development status of countries (developed vs. developing) levels in adherent patients (favours B) vs. non-adherent patients (favours A). The analysis includes 4 published papers [20,22,25,37] and the results of the present study [Bokayeva et al., 2025]; Figure S13: Subgroup meta-analysis of vitamin B12 levels according to development status of countries (developed vs. developing) levels in adherent patients (favours B) vs. non-adherent patients (favours A). The analysis includes four published papers [20,22,25,37] and the results of the present study [Bokayeva et al., 2025]; Figure S14: Subgroup meta-analysis of folate levels according to development status of countries (developed vs. developing) levels in regular patients (favours B) vs. irregular patients (favours A). The analysis includes two published papers [38,39] and the results of the present study [Bokayeva et al., 2025]; Figure S15: Subgroup meta-analysis of vitamin B12 levels according to development status of countries (developed vs. developing) levels in regular patients (favours B) vs. irregular patients (favours A). The analysis includes three published papers [21,38,39] and the results of the present study [Bokayeva et al., 2025]; PRISMA 2020 checklist.

## Abbreviations

The following abbreviations are used in this manuscript:

BH4	Tetrahydrobiopterin
BMI	Body Mass Index
CI	Confidence Interval
GRADE	Grading of Recommendations, Assessment, Development, and Evaluation
NA	Not Analysed
NI	No Information
NOS	Newcastle–Ottawa Scale
PAH	Phenylalanine Hydroxylase
Phe	Phenylalanine
PKU	Phenylketonuria
PRISMA	Preferred Reporting Items for Systematic Reviews and Meta-Analyses
SD	Standard Deviation
SMD	Standardised Mean Difference
STROBE	Strengthening the Reporting of Observational Studies in Epidemiology
Tyr	Tyrosine
