# Supplementary material for: Vitamin B12 and Folate in Adherent and Non-Adherent Individuals with Phenylketonuria: A Cross-Sectional Study, Systematic Review, and Meta-Analysis
Source: Metabolites. 2025 Jul 1;15(7):438. doi: 10.3390/metabo15070438 (PMC12298633; doi:10.3390/metabo15070438)
Supplement: Supplementary file 1 [file metabolites-15-00438-s001.zip › Table S1. Converted values for folate and B12 comparison.pdf]

Table S1. Comparison of vitamin status in studied individuals (with converted units).

Author	Year	Groups	Folate [1] [ng/mL]	Vitamin B12 [1] [pg/mL]
Bokayeva et al.	2025	Adherent	13.4 ± 1.96 [2]	767.6 ± 264.5 [2]
		Non-adherent	10.63 ± 3.36 [2]	524.7 ± 216.4 [2]
		Regular	13.32 ± 2.25 [2]	746.7 ± 228.4 [2]
		Irregular	10.48 ± 3.23 [2]	527.4 ± 281.9 [2]
Rojas-Agurto et al. [39]	2023	Regular [3]	25.69 ± 7.58 [2,5,6]	706.4 ± 330.4 [2,6]
		Irregular [4]	10.45 ± 3.17 [2,5,6]	383.4 ± 253.2 [2,6]
Akış et al. [25]	2020	Adherent [7]	16.42 ± 4.19 [2]	382.49 ± 174.17 [2]
		Non-Adherent [8]	15.58 ± 4.59 [2]	432.23 ± 166.98 [2]
Hochuli et al. [21]	2017	Regular [9]	98 ± 290 [2,5]	540 ± 208 [2]
		Irregular [10]	14 ± 3 [2,5]	251 ± 75 [2]
Gündüz et al. [22]	2016	Adherent [11]	14.30 ± 4.28 [2,5]	347.56 ± 188.75 [2]
		Non-Adherent [12]	14.47 ± 3.97 [2,5]	418.43 ± 161.38 [2]
Crujeiras et al. [37]	2015	Adherent	19.8 ± 7.0 [5,13]	749.6 ± 331.7 [13]
		Non-adherent	15.8 ± 8.6 [5,13]	515.9 ± 264.5 [13]
Schulpis et al. [20]	2002	Adherent [11]	1.04 ± 0.57 [2]	133.47 ± 30.22 [2]
		Non-adherent [12]	2.56 ± 0.93 [2]	326.29 ± 84.01 [2]

1—mean ± standard deviation; 2—serum; 3—patients under diet treatment; 4—patients who discontinued the protein substitution at 18 years of age; 5—folic acid; 6—data were received from authors; 7—patients with high adherence; 8—patients with low adherence; 9— regular AAM intake; 10 —AAM intake below the prescribed amount; 11—well-controlled; 12—poorly controlled; 13—plasma; for Robinson et al. [38] study the values was not recalculated due to its methodological issues.
