# Supplementary figures and images for: Vitamin B12 and Folate in Adherent and Non-Adherent Individuals with Phenylketonuria: A Cross-Sectional Study, Systematic Review, and Meta-Analysis
Source: Metabolites. 2025 Jul 1;15(7):438. doi: 10.3390/metabo15070438 (PMC12298633; doi:10.3390/metabo15070438)

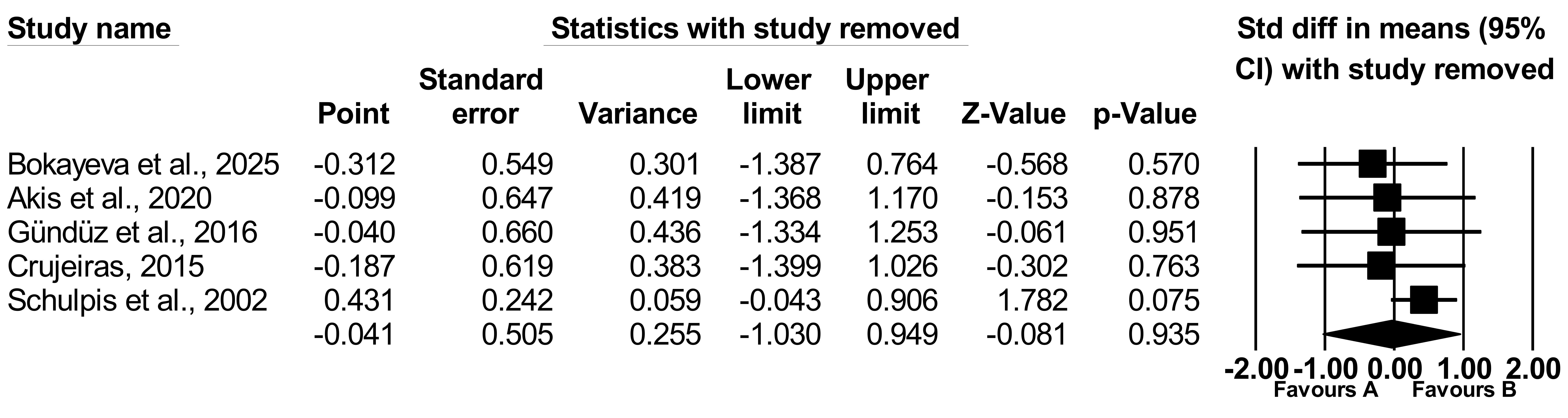

Supplement: Supplementary file 1 [file metabolites-15-00438-s001.zip › Figure S1. Sensitivity for folate (adherent vs non-adherent).jpg]

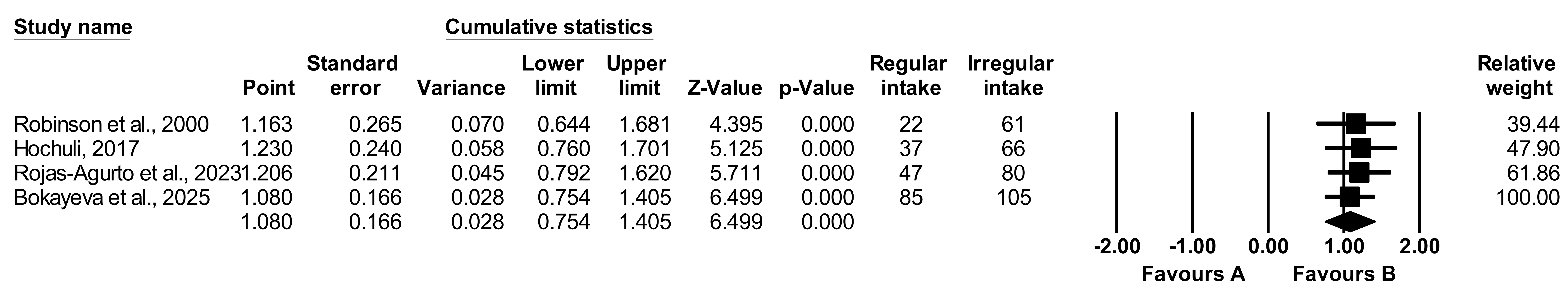

Supplement: Supplementary file 1 [file metabolites-15-00438-s001.zip › Figure S10. Cumulative for B12 (regular vs irregular).jpg]

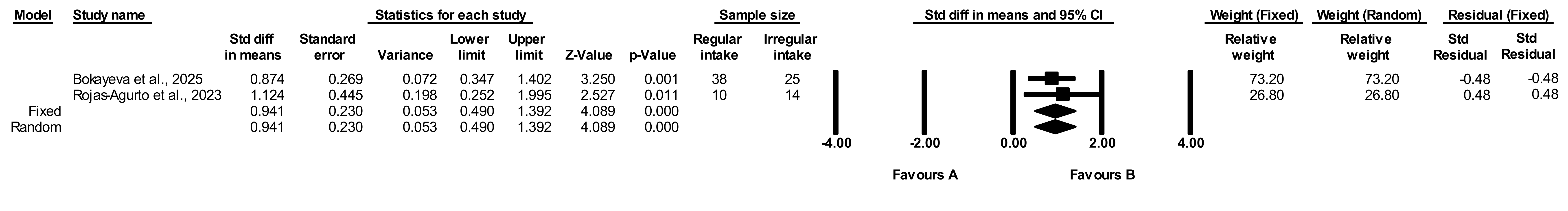

Supplement: Supplementary file 1 [file metabolites-15-00438-s001.zip › Figure S11. B12 forest plot without hrob (regular vs irregular).jpg]

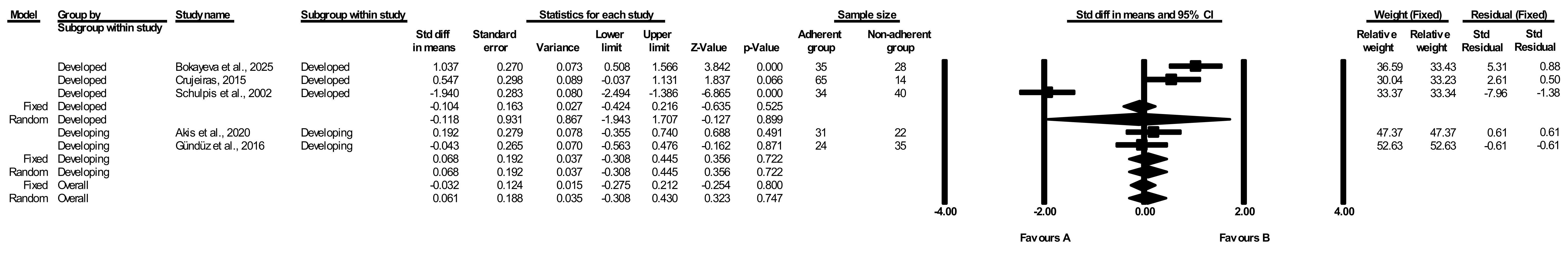

Supplement: Supplementary file 1 [file metabolites-15-00438-s001.zip › Figure S12. Folate forest plot - region subgroup (adherent vs non-adherent).jpg]

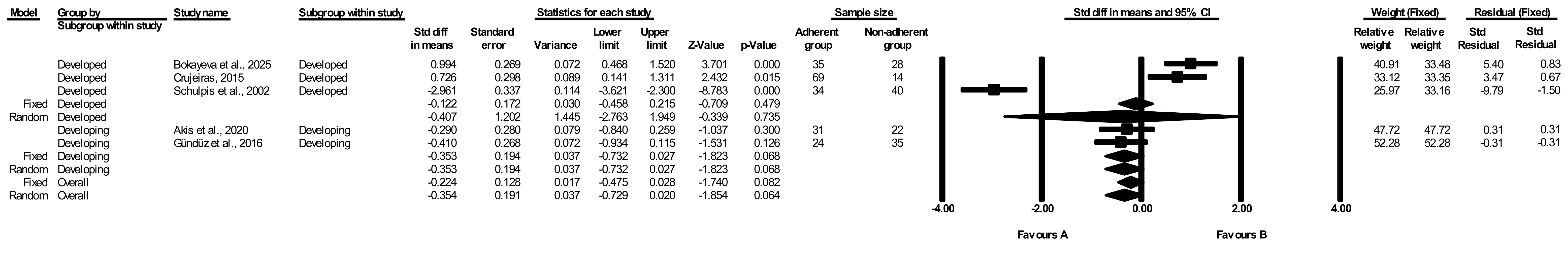

Supplement: Supplementary file 1 [file metabolites-15-00438-s001.zip › Figure S13. B12 forest plot - region subgroup (adherent vs non-adherent).jpg]

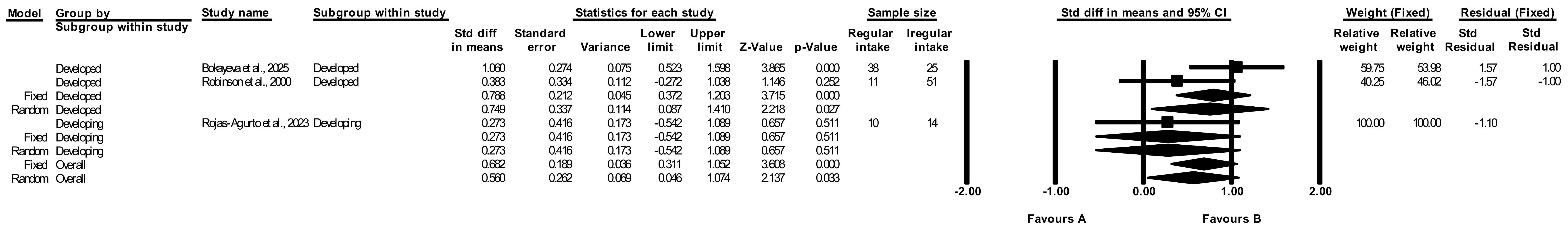

Supplement: Supplementary file 1 [file metabolites-15-00438-s001.zip › Figure S14. Folate forest plot - region subgroup (regular vs irregular).jpg]

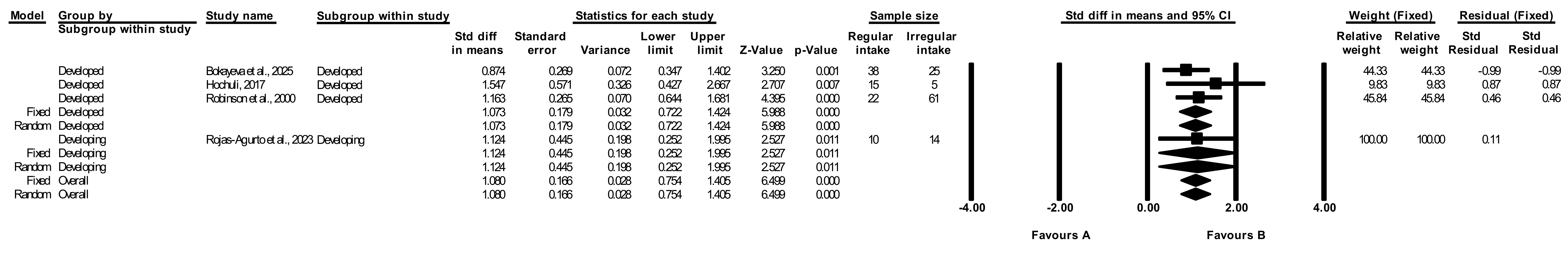

Supplement: Supplementary file 1 [file metabolites-15-00438-s001.zip › Figure S15. B12 forest plot - region subgroup (regular vs irregular).jpg]

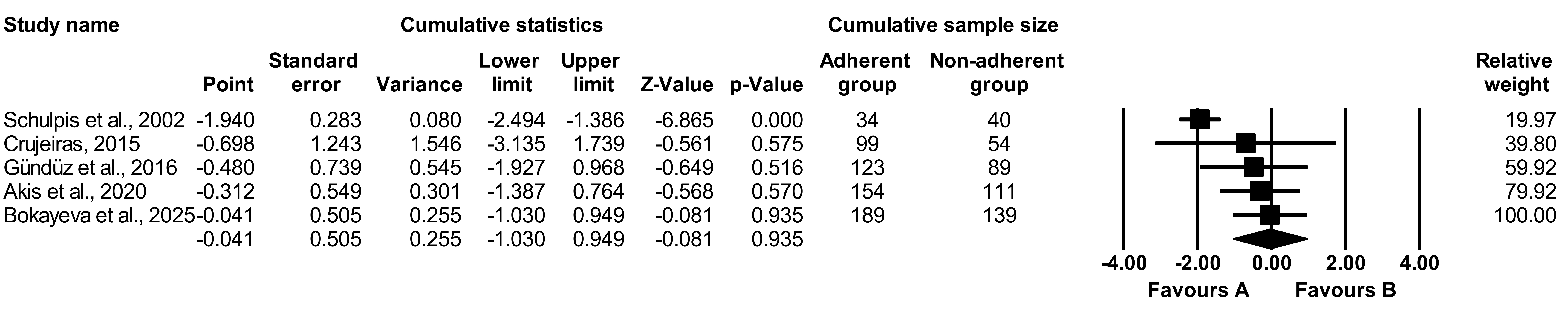

Supplement: Supplementary file 1 [file metabolites-15-00438-s001.zip › Figure S2. Cumulative for folate (adherent vs non-adherent).jpg]

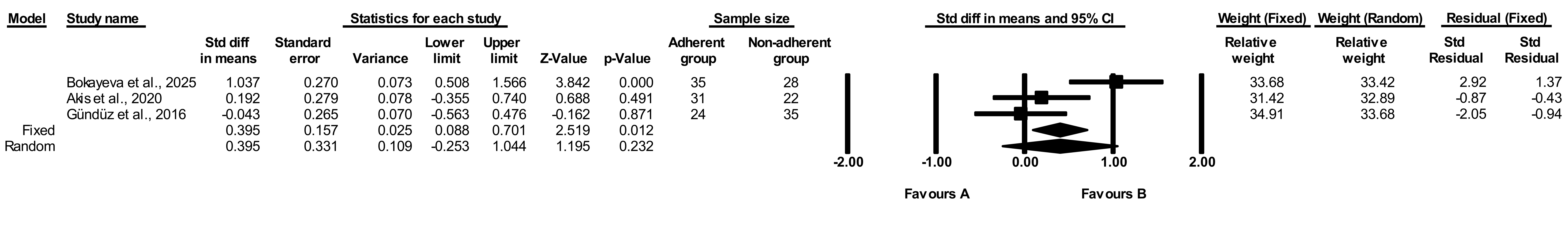

Supplement: Supplementary file 1 [file metabolites-15-00438-s001.zip › Figure S3. Folate forest plot without hrob (adherent vs non-adherent).jpg]

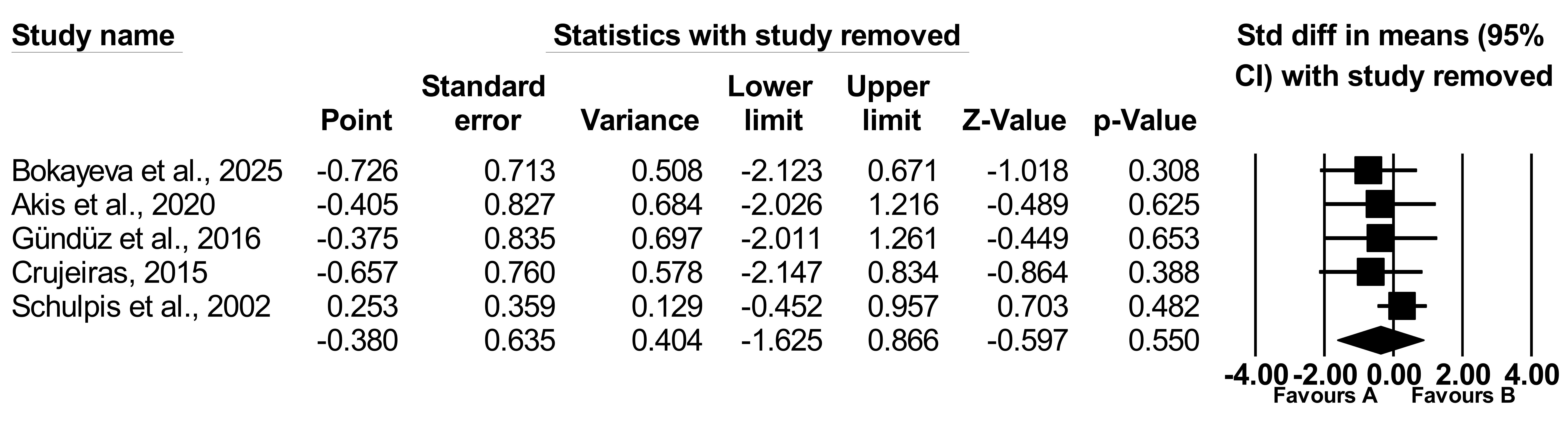

Supplement: Supplementary file 1 [file metabolites-15-00438-s001.zip › Figure S4. Sensitivity for B12 (adherent vs non-adherent).jpg]

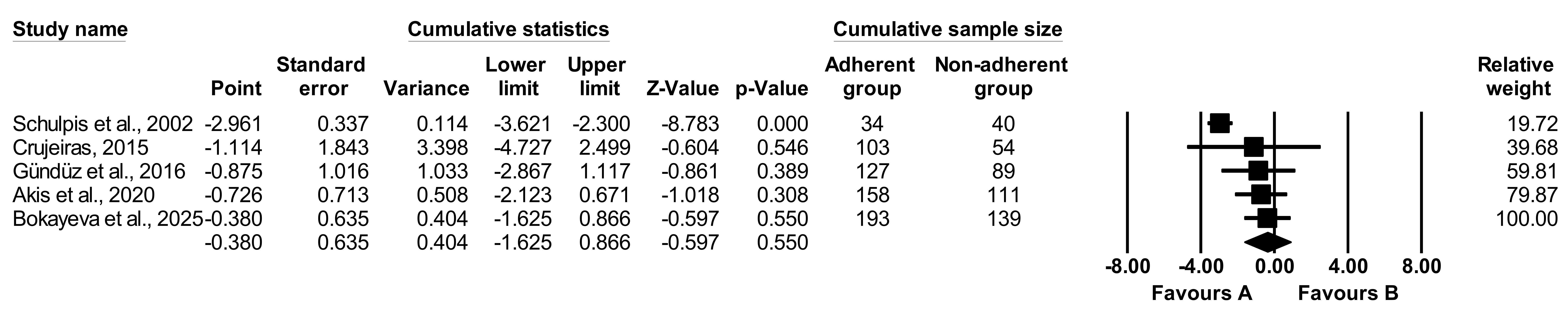

Supplement: Supplementary file 1 [file metabolites-15-00438-s001.zip › Figure S5. Cumulative for B12 (adherent vs non-adherent).jpg]

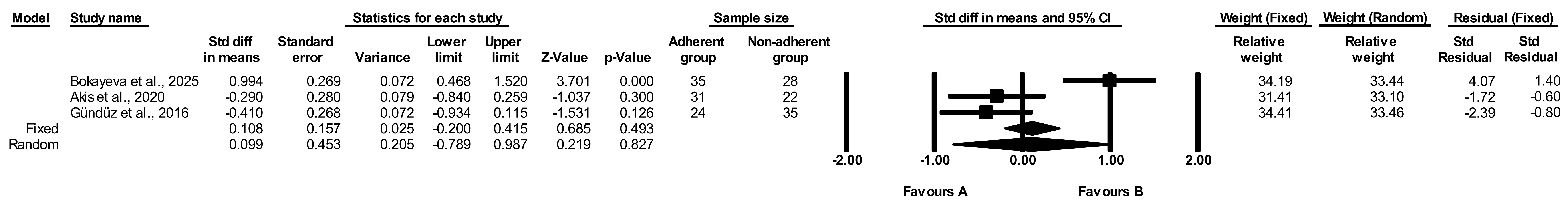

Supplement: Supplementary file 1 [file metabolites-15-00438-s001.zip › Figure S6. B12 forest plot without hrob (adherent vs non-adherent).jpg]

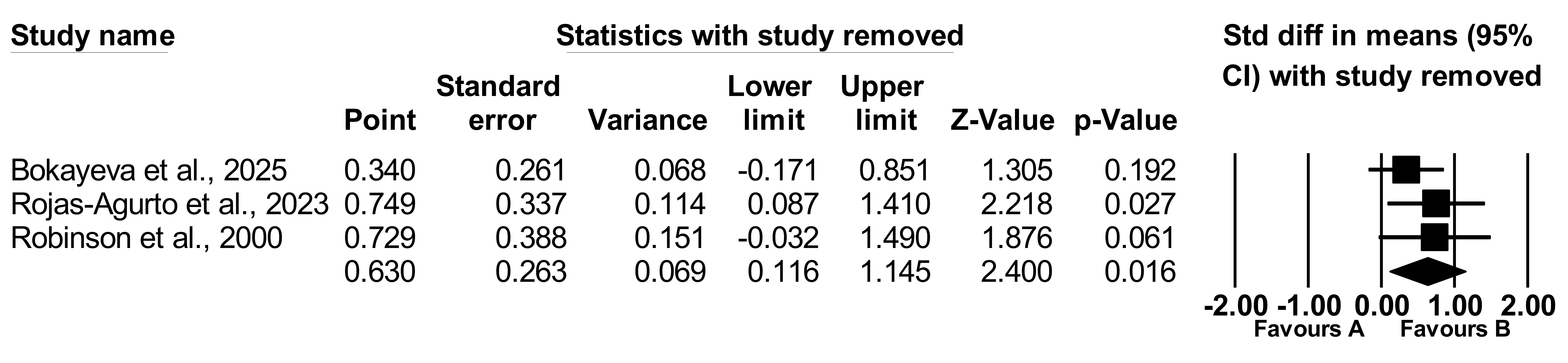

Supplement: Supplementary file 1 [file metabolites-15-00438-s001.zip › Figure S7. Sensitivity for folate (regular vs irregular).jpg]

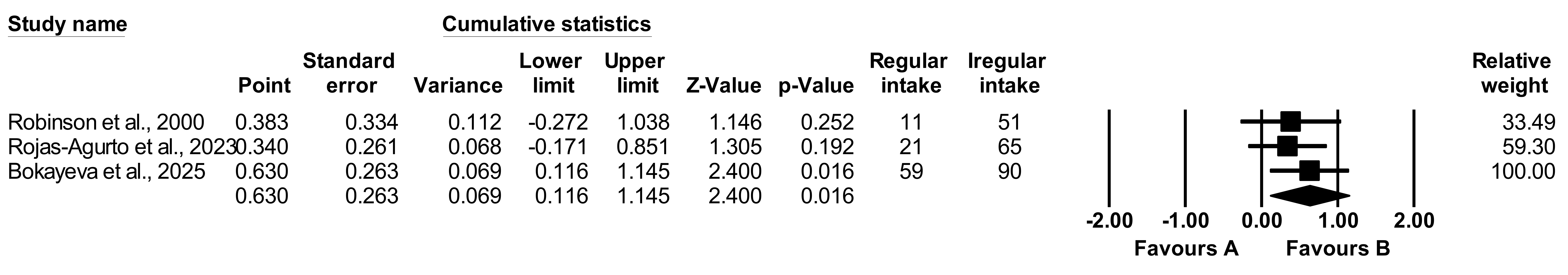

Supplement: Supplementary file 1 [file metabolites-15-00438-s001.zip › Figure S8. Cumulative for folate (regular vs irregular).jpg]

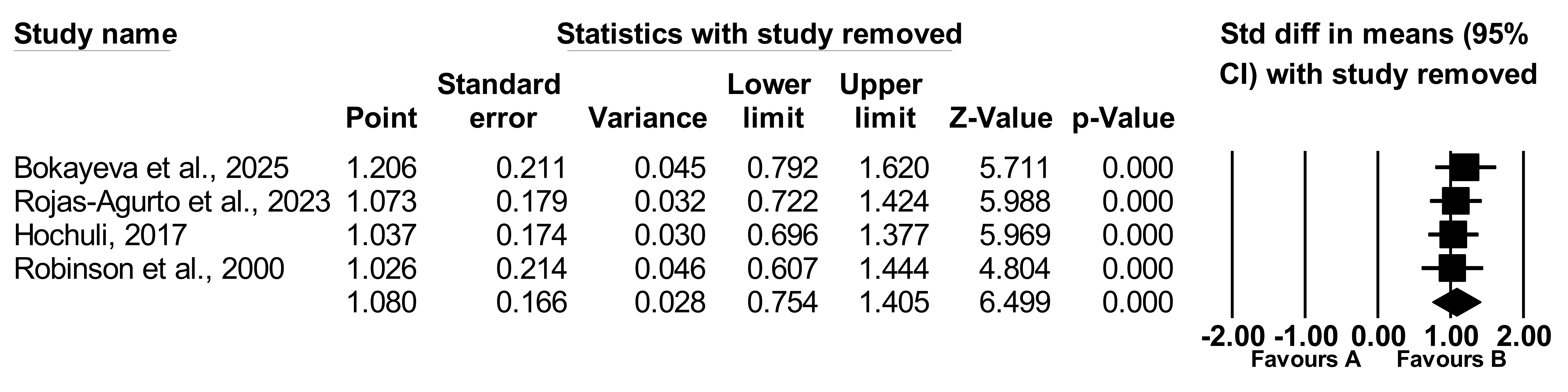

Supplement: Supplementary file 1 [file metabolites-15-00438-s001.zip › Figure S9. Sensitivity for B12 (regular vs irregular).jpg]
